# Four New Bat Species (*Rhinolophus hildebrandtii* Complex) Reflect Plio-Pleistocene Divergence of Dwarfs and Giants across an Afromontane Archipelago

**DOI:** 10.1371/journal.pone.0041744

**Published:** 2012-09-12

**Authors:** Peter J. Taylor, Samantha Stoffberg, Ara Monadjem, Martinus Corrie Schoeman, Julian Bayliss, Fenton P. D. Cotterill

**Affiliations:** 1 Department of Ecology and Resource Management, University of Venda, Thohoyandou, South Africa; 2 Durban Natural Science Museum, Durban, South Africa; 3 Evolutionary Genomics Group, Department of Botany and Zoology, University of Stellenbosch, Stellenbosch, South Africa; 4 All Out Africa Research Unit, Department of Biological Sciences, University of Swaziland, Kwaluseni, Swaziland; 5 School of Life Sciences, University of KwaZulu-Natal, Durban, South Africa; 6 Mulanje Mountain Conservation Trust, Mulanje, Malawi; 7 Conservation Science Group, Department of Zoology, University of Cambridge, Cambridge, United Kingdom; 8 Africa Earth Observatory Network, Geoecodynamics Research Hub, Department of Botany and Zoology, University of Stellenbosch, Stellenbosch, South Africa; BiK-F Biodiversity and Climate Research Center, Germany

## Abstract

Gigantism and dwarfism evolve in vertebrates restricted to islands. We describe four new species in the *Rhinolophus hildebrandtii* species-complex of horseshoe bats, whose evolution has entailed adaptive shifts in body size. We postulate that vicissitudes of palaeoenvironments resulted in gigantism and dwarfism in habitat islands fragmented across eastern and southern Africa. Mitochondrial and nuclear DNA sequences recovered two clades of *R. hildebrandtii* senso lato which are paraphyletic with respect to a third lineage (*R. eloquens*). Lineages differ by 7.7 to 9.0% in cytochrome b sequences. Clade 1 includes *R. hildebrandtii* sensu stricto from the east African highlands and three additional vicariants that speciated across an Afromontane archipelago through the Plio-Pleistocene, extending from the Kenyan Highlands through the Eastern Arc, northern Mozambique and the Zambezi Escarpment to the eastern Great Escarpment of South Africa. Clade 2 comprises one species confined to lowland savanna habitats (Mozambique and Zimbabwe). A third clade comprises *R. eloquens* from East Africa. Speciation within Clade 1 is associated with fixed differences in echolocation call frequency, and cranial shape and size in populations isolated since the late Pliocene (*ca* 3.74 Mya). Relative to the intermediate-sized savanna population (Clade 2), these island-populations within Clade 1 are characterised by either gigantism (South African eastern Great Escarpment and Mts Mabu and Inago in Mozambique) or dwarfism (Lutope-Ngolangola Gorge, Zimbabwe and Soutpansberg Mountains, South Africa). Sympatry between divergent clades (Clade 1 and Clade 2) at Lutope-Ngolangola Gorge (NW Zimbabwe) is attributed to recent range expansions. We propose an “Allometric Speciation Hypothesis”, which attributes the evolution of this species complex of bats to divergence in constant frequency (CF) sonar calls. The origin of species-specific peak frequencies (overall range = 32 to 46 kHz) represents the allometric effect of adaptive divergence in skull size, represented in the evolution of gigantism and dwarfism in habitat islands.

## Introduction

According to the “Island rule”, adaptation to insular environments results in dwarfism in larger mammals such as ungulates and carnivores, in contrast to gigantism in smaller mammals such as rodents [1]. Although the universality of this rule in vertebrates has been debated [2], [3], [4], several studies of Chiroptera have shown a tendency for island species and subspecies to be considerably smaller than continental relatives [5], [6], [7], [8]. Although these empirical studies have focussed on bats on true islands, the rule was hypothesized to also apply to habitat islands. For example, significant historical changes in body length of European mammals over 175 years is attributed to recent habitat fragmentation [9].

**Figure 1:**
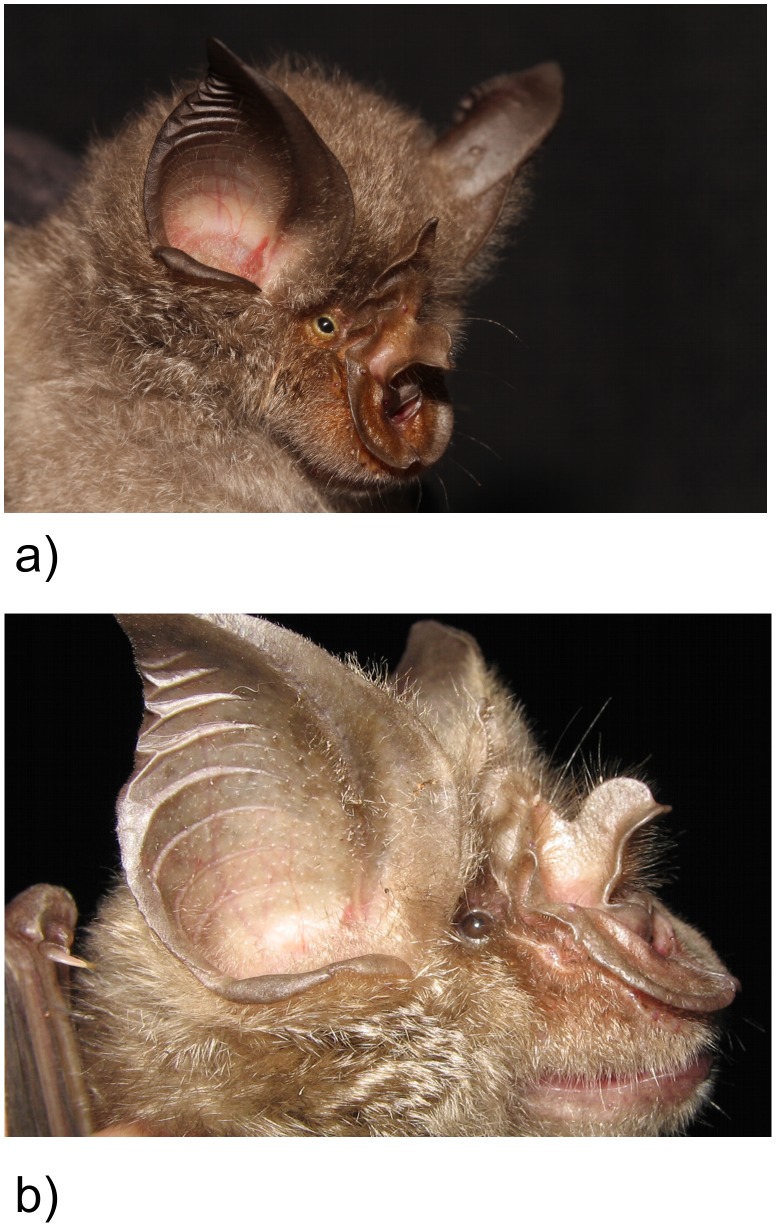
Portraits of (a) *Rhinolophus smithersi* species novo, and (b) *Rhinolophus mossambicus* species novo, two of four new cryptic species described herein within the *R. hildebrandtii* complex.

This paper presents the findings of a multi-disciplinary taxonomic study of *Rhinolophus hildebrandtii*, initially motivated by the discovery of distinct sonotypes in southern Africa within an apparently monotypic species of horseshoe bat. Horseshoe bats (Genus *Rhinolophus* Lacépède 1799) are the only extant members of the family Rhinolophidae (Bell 1836) and their distribution is restricted to the Old World. All members of this monogeneric family are characterized by a horseshoe-shaped anterior noseleaf (Fig. 1) and the different parts (lancet, connecting process, sella and noseleaf) often differ in size and shape and can be diagnostic in identifying species. Other characters often used in discriminating rhinolophid species include forearm length, craniodental measurements, the presence (or absence) and position of the anterior upper premolar, the number of mental grooves in the lower lip and peak echolocation frequency of the CF component. However, in many instances there may be overlap in one or more of these characters making molecular analysis an important tool in confirming species identities.

Hildebrandt's Horseshoe Bat (*Rhinolophus hildebrandtii* Peters, 1878; Fig. 1) is a large horseshoe bat (forearm length 60–67 mm) distributed across the Afromontane Archipelago of Africa [10], [11] and easily distinguished from other African horseshoe bats on size alone [12], [13]. Similar, but slightly smaller species include the Eloquent Horseshoe Bat (*R. eloquens* Anderson, 1905) and Rüppell's Horseshoe Bat (*R. fumigatus* Rüppell, 1842). All three species belong to the *fumigatus*-group [12], are endemic to the Afrotropical zoogeographic region, and overlap in forearm length. Only *R. eloquens* and *R. hildebrandtii* overlap in craniodental measurements [12] and both species possess a single mental groove in the lower lip and long hairs on the sella [12]. Although the distributions of both species overlap in Kenya, Rwanda, Sudan, Tanzania, Uganda and Zaire [12], that of *R. eloquens* does not extend into southern Africa whereas *R. hildebrandtii* is described from DRC, Botswana, Zimbabwe, Malawi, Zambia, Mozambique and the northern parts of South Africa [13], [14]. *Rhinolophus hildebrandtii* (33–46 kHz [13]) and *R. fumigatus* (53 kHz [13]) can be distinguished using echolocation call frequency; that of *R. eloquens* is unknown.

Echolocation frequency is often used to identify bats, especially morphologically similar species, and has helped to reveal cryptic species [15]. Horseshoe bats are high-duty cycle echolocators (long call duration relative to the interval between consecutive calls) and have calls that are dominated by a long constant-frequency (CF) component. Growing evidence suggests that *R. hildebrandtii* as currently recognised is a species complex, which is not necessarily monophyletic [13], [16]. Several discrete sonotypes exist with peak frequencies at 42 kHz (from Masai Mara National Reserve to Taita-Kasegau Wildlife Corridor in Kenya), both 37 and 46 kHz (occurring sympatrically at the Lutope-Ngolangola Gorge in NW Zimbabwe), 35–37 kHz (at several localities in Mozambique), 44 kHz (at Pafuri in the Kruger National Park in the extreme north of South Africa) and 33 kHz (at Sudwala and Barberton in the Mpumalanga Province in the northeast of South Africa) (Table S1). Similarly, two distinct sonotypes within *R. hildebrandtii* have been recorded in Mozambique that are also morphologically distinct [13]. Although peak frequency of the CF component in rhinolophid bats can vary with geography, sex or age [16], acoustic divergence (variation in peak frequency) has been associated with speciation in *Rhinolophus* [17] and in species in the sister family of bats, the Hipposideridae [17], [19] and peak frequency is strongly associated with species recognition and the facilitation of intraspecific communication [20].

**Figure 2:**
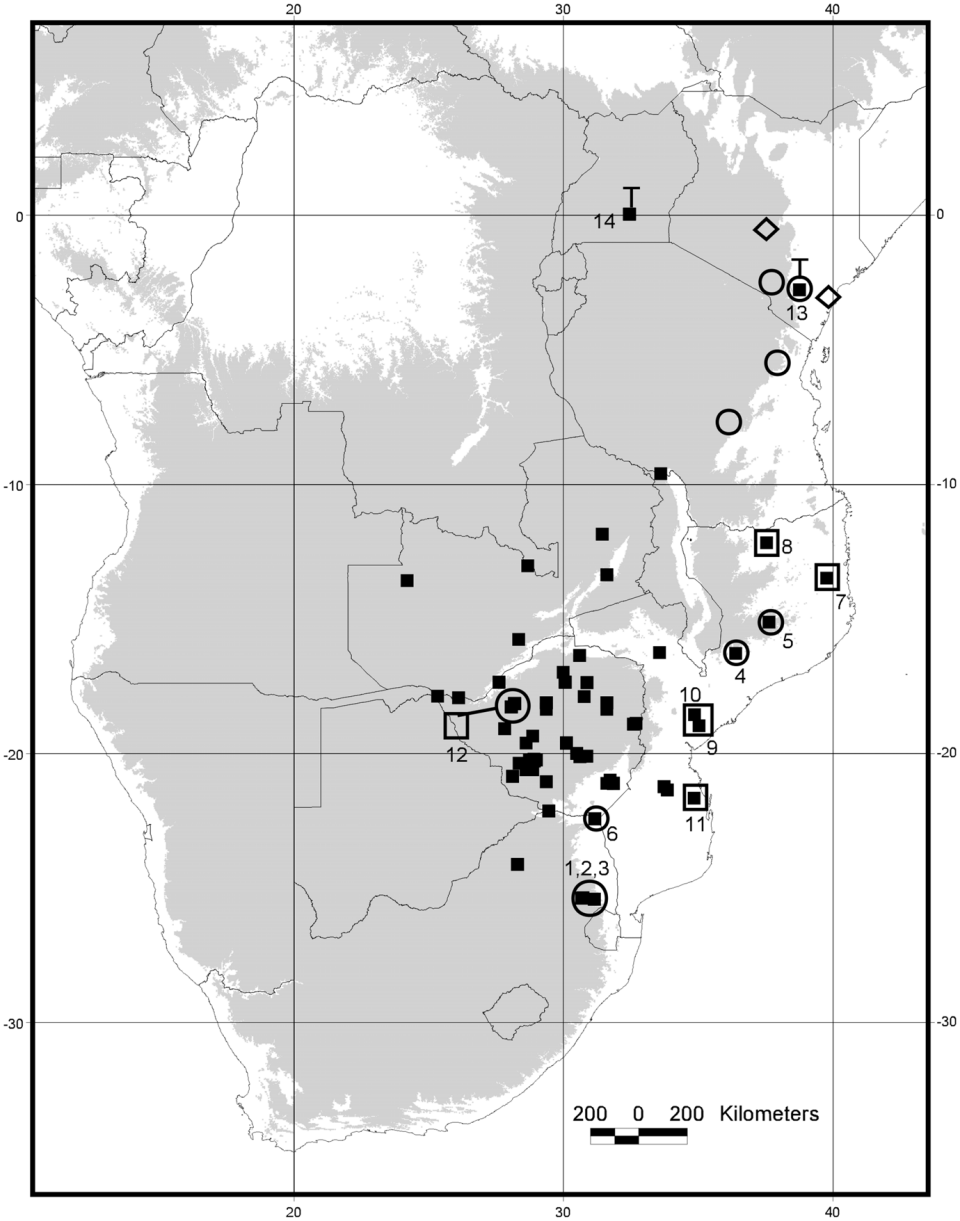
Map of southern, central and eastern Africa indicating localities of individuals of *R. hildebrandtii* species-complex included in this study. Grey-shaded area represents elevations in excess of 600 m a.s.l. Closed squares indicate museum specimens from which craniometric data were obtained. Open symbols indicate specimens genotyped in this study. The distribution of the three major clades is based on cytochrome b (see Figure 2): open circles = Clade 1; open squares = Clade 2; open diamonds = Clade 3. Closed squares enclosed in open symbols indicate localities where both molecular and morphological data were available for selected specimens. Numbers refer to respective localities listed in Table S1. “T” indicates the type localities of *R. eloquens* in Uganda and *R. hildebrandtii* in Kenya, respectively.

The aim of this study was to resolve the phylogenetic relationships and species limits of *R. hildebrandtii* using a multidisciplinary approach (DNA sequences, acoustic data and cranial, noseleaf and bacular morphology) based on recent collections (Fig. 2; Table S1) from Mozambique, South Africa and Zimbabwe. In order to test monophyly and to accurately delimit the species boundaries, we included *R. eloquens* (the species morphologically most similar to *R. hildebrandtii*) in our analyses. We included samples of *R. hildebrandtii* from the Taita-Taveta District, Kenya which is the type locality for *R. hildebrandtii*, in the molecular analyses and included skull measurements from type series of both *R. hildebrandtii* (from Ndi, Taita District, Kenya) and *R. eloquens* (from Entebbe, Uganda). Finally we discuss the adaptive nature of patterns of variation in echolocation frequency, body size and cranial morphology.

## Materials and Methods

### Collection of samples

Tissue samples and morphological descriptions and measurements came both from historical museum collections, as well as from more recent biodiversity surveys which have been reported elsewhere [13], [16], [21], [22] (Table S1). Specimens from Zimbabwe (reported in 13,16) were collected during routine surveys conducted by FPDC under the authority of (and employed by) the National Museums of Zimbabwe and did not require permits. Specimens from Mozambique were collected by JB [22] and AM [21] with permission from Instituto de Investigação Agrária de Moçambique (IIAM), for unprotected areas, which also required the permission of landowners, and the Sociedade para a Gestão e Desenvolvimento da Reserva Do Niassa, Maputo (SRN) for a formally protected area (permission letter to AM dated 6 September 2006). Voucher specimens were deposited as required both in the Museu de História Natural, Maputo as well as the Durban Natural Science Museum. Specimens from South Africa were collected with the permission and cooperation from the Mpumalanga Tourism and Parks Agency (MTPA) and from local landowners; collecting was conducted on private land (no permit needed) as well as a formally protected area under the management of the MTPA (P1 permit number MPB 8621) and voucher specimens were deposited as required in the Durban Natural Science Museum.

### Molecular systematics

#### Sampling, DNA extraction and nucleotide sequencing

Tissue samples or wing biopsy punches from 37 individuals (Table S1) were sequenced. Sequenced taxa include 27 representatives of *R. hildebrandtii sensu lato* (based on keys in [12]) from Kenya, Mozambique, South Africa, Tanzania and Zimbabwe; five individuals of *R. eloquens* from Kenya; and five individuals of *R. clivosus* from Mozambique and South Africa. Total genomic DNA was extracted using either the Wizard® SV Genomic DNA Purification System (Promega, Madison, Wisconsin, USA) or the DNeasy Blood and Tissue Kit (Qiagen, Hilden, Germany) following manufacturer's recommendations.

Three mitochondrial DNA (mtDNA) gene fragments were amplified: 1) a section of the control region (N777 and DLH1; [23]); 2) a section of the 12S rRNA gene (12S AL and 12S BH; [24] and 3) a 535 bp section of the cytochrome b gene using the primers RhinoCytbF 5′ CTA CCC TTC ATT ATC GCA GC 3′ and RhinoCytbR 5′ CCT GCA AGT GGT ATT AGG AC 3′ (J. M. Bishop, unpublished). We also amplified a section of the nuclear intron from the nucleosome remodelling factor gene (Chd1) using the primers and polymerase chain reaction (PCR) thermal profile described in [25]. PCR thermal conditions for mtDNA genes were an initial 5 min denaturation at 95°C, followed by 35 cycles of 30 s at 94°C, 45 s annealing at 50°C (54°C for cytochrome b), 45 s at 72°C, and a final extension cycle at 72°C for 10 min. All PCR reactions included a negative (all reagents, but no template) to check for possible contamination. A subsample of PCR products were visualized on 1.0% agarose gel containing ethidium bromide. The remaining product was sent to the Core Sequencing Facility, Stellenbosch University, South Africa, where the PCR products were cleaned and cycle sequencing performed using BigDye chemistry (Applied Biosystems, Perkin Elmer). Sequencing products were then analysed on an ABI 3100 (Applied Biosystems, Perkin Elmer) automated sequencer.

#### Sequence Analyses

Chromatograms were visualized and aligned using BioEdit v7.0.1 [26]. The resultant datasets comprised: 1) control region - 33 taxa and 525 bp (101 bp parsimony-informative); 2)12S - 27 taxa and 368 bp (23 bp parsimony-informative); 3) cytochrome b - 20 taxa and 535 bp; and 4) Chd1 - 20 taxa and 778 bp (15 bp parsimony-informative). Sequences were deposited in GenBank under the following accession numbers: JQ929202–JQ929301. Not all individuals would amplify for all gene fragments resulting in many missing sequences. Thus the datasets were not combined, but analysed separately. Phylogenetic hypotheses were estimated using both parsimony and Bayesian analyses for the control region, 12S and Chd1 data sets. Parsimony analysis was done using the heuristic search option, with all site changes weighted equally [27] in PAUP* 4.0b10 [28]. Tree-bisection-reconnection (TBR) branch swapping was used and starting trees were obtained via 100 random stepwise additions. Bootstrap support [29] was calculated using 1000 replicates. Calculation of uncorrected (not based on a particular model of evolution) pairwise genetic distances for each gene was conducted using PAUP* 4.0b10.

The most appropriate model of molecular evolution was determined for each gene fragment using the Akaike Information Criterion (AIC) and Bayesian Information Criterion (BIC) as implemented in jModelTest 0.1.1 [30]. The model parameters for each dataset were used in subsequent analyses.

Bayesian analyses (BA) were conducted using Mr Bayes 3.1.2 [31]. Four incrementally heated chains were run for three million generations, with parameters sampled every 1000 generations. Convergence of the MCMC chains was assessed by inspecting whether the standard deviation of split frequencies approached zero and the potential scale reduction factor (PSRF) reached 1.0 for all parameters. We also investigated the convergence using Tracer v 1.4.1 [32]. A 25% burnin was used and the 50% majority rule consensus tree was constructed from the remaining tree data.

#### Molecular dating

To estimate divergence dates, the cytochrome b data set was analysed in BEAST 1.4 [29]. We included additional *R. hildebrandtii* sequences from Pafuri [33] and Tanzania (EU436676, [34]) as well as other African *Rhinolophus* species downloaded from GenBank: *R. darlingi* (EU436675); *R. eloquens* (EU436677); *R. fumigatus* (FJ457614); *R. landeri* (EU436668, FJ457612) and *R. ruwenzorii* (EU436679 and FJ185203). In order to be able to use fossil calibration points we incorporated sequences downloaded from GenBank for six species in the family Hipposideridae, sister to the Rhinolophidae: *H. armiger* (DQ865345), *H. caffer* (EU934461), *H. cyclops* (EU934466), *H. gigas* (EU934469), *H. pratti* (EF544427) and *H. ruber* (EU934485). jModelTest [30] was used to determine the most appropriate model of evolution. BEAUTi was used to set model parameters and the monophyly of the ingroup (*Rhinolophus*) was constrained during analysis. We used the HKY model with empirical base frequencies and the substitution rate was not fixed. The Yule speciation process was used as the tree prior and a relaxed uncorrelated lognormal molecular clock model was selected. A normal distribution for the tree prior for the node delimiting time to the most recent common ancestor was selected. As calibration points we followed Teeling *et al.* (2003) and Eick *et al.* (2005) and used a minimum of 37 Mya and maximum of 55Mya for the split between the Rhinolophidae and the Hipposideridae [35], [36]. The MCMC chain was run for 20 million generations, with parameters logged every 1000 generations.

Results were evaluated using Tracer v1.4.1 [32]. The Effective Sample Size (ESS) values were >200 for all parameters, suggesting the MCMC run was sufficient and independent samples were incorporated to obtain valid parameter estimates [32]. Trees were collated using TreeAnnotator 1.6 where mean heights and a burnin of 10% were selected.

### Acoustic recordings and an analysis of echolocation frequency predictors

Values for peak frequency (which in this study includes ‘maximum’ frequency from frequency-division bat detectors) of the CF component of echolocation calls of *R. hildebrandtii* were obtained from the literature [13], [16], [37], [38]. Additional recordings were obtained from Mozambique, South Africa and Zimbabwe (Tables S1 and S2) using an ANABAT II bat detector (Titley Electronics, Ballina, Australia), Avisoft UltraSoundGate 116 (Avisoft Bioacoustics, Berlin, Germany) or a Pettersson D240× or D980 bat detector (Pettersson Electronik AB, Uppsala, Sweden). Echolocation recordings from ANABAT recordings were analysed with ANALOOK (Chris Corben, version 4.8), and Pettersson recordings with either Raven Pro version 1.3 (R. A. Charif, A. M. Waack, and L. M. Strickman — Cornell Laboratory of Ornithology), or with BatSound Pro v3.20 (Pettersson Electronik AB, Uppsala, Sweden) software. For ANABAT recordings, we defined peak echolocation frequency as F(max) [39]. For time expansion recordings, peak frequency was measured from the peak of the power spectrum [40]. It was assumed that values of peak frequency determined with time-expansion detectors [16], [38] and frequency division detectors (i.e. ANABAT detector; [21]; Monadjem, unpublished data) would be close enough to make meaningful comparisons for the purpose of the present study.

We developed linear models in R2.13.1 (R Development Core Team, Vienna; http://www.r-project.org) to test the significance of various predictors of the peak frequency of the echolocation call. Based on a sample of 36 individuals with known frequency (specimens with recorded frequencies listed in Table S1, excluding the two Kenyan *hildebrandtii* s.s. individuals), we tested up to ten independent variables in R with hierarchical nested linear models of increasing complexity. The predictor variables included forearm length, five cranial variables (defined below: CCL, ZYW, NL, NW, NH), two environmental variables, altitude (obtained from www.worldclim.org) and relative humidity (obtained from U.S. National Aeronautics and Space Administration (NASA), Surface meteorology and Solar Energy (SSE) programme, release 6.0: http://eosweb.larc.nasa.gov/sse/), and two categorical variables, sex and molecular clade membership (Clade 1 or Clade 2). The simplest models included just skull length (CCL) and altitude. Because of the potential effect of altitude on body (and therefore cranial) size (Bergman's Rule) we included altitude-group (lowland <600 m; highland >600 m) as a co-variate both with (CCL*Altitude; Model 1) and without (CCL+Altitude, Model 2) interaction. The interaction term for CCL and altitude in Model 1 (β = −0.7402) was not significant (p>0.05), and the Log-Likelihood Ratio test did not find a significant difference between Models 1 and 2. Thus, later models did not allow interaction. Model 3 was similar to Model 2 but used altitude as a continuous rather than categorical variable. Based on Akaike Information Criteria (AIC) values, Model 3 was inferior to Model 2 and thus the categorical variable for altitude performed better and was used in later models. Model 4 included all five cranial variables, forearm length and the two environmental variables mentioned above (altitude and relative humidity). Model 5 included all these variables in addition to two additional categorical variables, sex and clade. Model fit of each model was assessed inter alia by inspecting plots of residuals for fit to normal distribution. Relative performance of models was assessed using likelihood ratio tests and AIC values.

### Morphology

#### Specimens

Since our objective was to compare multiple datasets (molecular, acoustic and morphological) for the same critical samples, we focussed on recent collections of skins and skulls for which molecular and acoustic data were available (localities indicated by open symbols in Fig. 2) from Zimbabwe, South Africa and Mozambique in the collections of the National Museums of Zimbabwe (NMZB; Bulawayo), Durban Natural Science Museum (DM) and Ditsong National Natural History Museum (formerly Transvaal Museum; TM) (Table S1; Fig. 2). An additional seven skulls were added to the analysis, representing type series of *R. eloquens* (n = 5; Natural History Museum, London; BM) and *R. hildebrandtii* (n = 2; Zoologisches Museum, Berlin; ZMB). One additional damaged skull of the co-type from the Natural History Museum in London (BM) was examined but not included in the final morphometric analysis. We had access to a database of cranial measurements from a much larger collection of both *eloquens* and *hildebrandtii* from the following museum collections: DM, Harrison Zoological Museum (HZM; Sevenoaks, UK), NMZB, BM, ZMB and TM. Since preliminary principal component analyses (PCA) showed the Zimbabwe *R. hildebrandtii* sample to be relatively homogenous craniometrically, we used a sample of 109 *R. hildebrandtii* skulls from Zimbabwe to test for the presence of significant sexual dimorphism in this species.

To investigate further the species limits and morphological diagnosability of lineages defined by genetic and acoustic data, we analysed variation in the larger sample of 255 intact skulls using six craniometric variables (GLS, CCL, ZW, MW, M3M3, CM3; see definitions below). Since preliminary analyses of non-geographic variation (available from PJT) detected significant sexual dimorphism, males and females were analysed separately and only those for males (n = 171) were presented. Samples were grouped to pool biogeographically-similar localities into Operational Taxonomic Units (OTUs). Exploratory PCA analyses of individuals were used to test morphological homogeneity of defined OTUs. Due to scarcity of available samples, specimens from East Africa were grouped into single OTUs (for both *R. eloquens* and *R. hildebrandtii*) and specimens of *R. hildebrandtii* from Zambia and Malawi were combined. Specimens of *R. hildebrandtii* from Zimbabwe were grouped into four OTUs (north, central, east and south). Specimens from lowland savanna localities in Mozambique (Tete and Zinave) were assigned to the biogeographically continuous “south” OTU from the southeastern “lowveld” of Zimbabwe. Other Mozambican and South African localities for which molecular data were available were assigned to either Clade 1 or Clade 2 or to lineages as defined by molecular analysis (see Results).

#### Morphological and morphometric analyses

In additional to morphometric analysis of continuous characters, we scored the following qualititative, craniodental characters: the presence, position (external or within toothrow) and relative size (small or “tiny”) of the small anterior upper premolar and the relative height of the anterior nasal swelling and sagittal crest in lateral view.

For morphometric analysis, adult rhinolophids were selected based on degree of tooth wear, and extent of ossification of epiphyses in the finger bones. We used two morphometric approaches: analysis of traditional linear measurements as well as landmarks placed on dorsal and lateral images of crania.The following 12 cranial measurements were taken to the nearest 0.01 mm using Mitutoyo digital callipers with accuracy of 0.01 mm: greatest length of skull measured dorsally from occiput to anterior point of skull (GSL); condylo-incisive length from occipital condyles to front of incisors (CIL); condylocanine length from occipital condyles to front of canines (CCL); zygomatic width, the greatest distance across the zygoma (ZW); mastoid width, the greatest distance across the lateral projections of the mastoid processes (MW); width of maxilla between outer edges of M3 (M3M3); braincase width measured at dorsal surface of posterior root of zygomatic arches (BCW); least interorbital width between orbits (IOW); upper toothrow length from anterior surface of C to posterior surface of M3 (CM3); greatest width across anterior lateral nasal inflations (NW); length from occipital condyles to front of nasal inflations (NL); and height of nasal inflation directly above the anterior cingulum of M2 (NH) [12], [41]. Principal component analysis (PCA) and canonical variates analysis (CVA) was carried out on log-transformed variables using the programme XLSTAT version 2008.2.03 [42].

A Sony Cybershot DSC-H2 digital camera (6 megapixel; 12× optical zoom and ×2 converter; macro function), mounted on a tripod at a fixed distance of 20 cm from the skull (which was always mounted on graph paper), was used to take dorsal and lateral images for 22 skulls from the same sample used for linear measurements.

Landmark placement and further analyses were performed using the thin plate spline (TPS) series of programmes. The programme tpsDig version 2.1 [43] was used to capture landmarks in two dimensions for dorsal (13 landmarks) and lateral (12 landmarks) views (see Figs. 6 and 7 for position of landmarks on lateral and dorsal images respectively). The programme tpsRelw version 1.42 [44] was used to conduct a Generalised Procrustes Analysis or GPA (Generalised Least Squares, GLS, [45]), which serves to translate, rotate and scale the landmark configurations, and produces a consensus configuration for the entire suite of specimens in the analysis via a series of iterations. GPA residuals are further decomposed into both non-uniform (non-affine), and uniform (affine) shape components. Non-affine shape expresses localized shape changes, and is represented by the weights matrix, W, of partial warp scores. Affine shape expresses shape changes that affect the entire configuration (i.e. dilation or sheer), and this component is represented by two vectors, U1 and U2. Together, U+W represent total shape. Relative warps analysis performs a PCA of the covariance matrix of the total shape matrix (U+W).

For introductions to geometric morphometrics and its application to mammalian systematics see [46], [47], [48], [49], [50].

#### Morphology of noseleaf

Noseleafs of alcohol-preserved specimens were photographed in lateral and frontal views. Maximum noseleaf width was measured in 44 specimens included in this study.

#### Morphology of baculum

Preparation of 12 *R. hildebrandtii* bacula from males from six localities (Niassa Game Reserve, Gorongosa Caves and Mt Mabu in Mozambique, and Barberton Tunnels, Sudwala Mines and Mayo Mines in Mpumalanga Province of South Africa) followed standard procedures [51], [52], [53]: penial tissue was macerated in 5% KOH and the baculum stained with alizarin red followed by dissection of the baculum and clearing with glycerine. Bacula were stored in 100% glycerine with a crystal of thymol to prevent fungal growth. Each baculum was photographed in dorsal, ventral and lateral view and total baculum length (TBL), measured along the axis of the shaft, was recorded using Mitutoyo digital callipers viewed under a dissecting microscope. All bacular photographs were taken against a background of graph paper to facilitate scale drawings of the bacula.

### Nomenclatural acts

The electronic version of this document does not represent a published work according to the International Code of Zoological Nomenclature (ICZN), and hence the nomenclatural acts contained in the electronic version are not available under that Code from the electronic edition. Therefore, a separate edition of this document was produced by a method that assures numerous identical and durable copies, and those copies were simultaneously obtainable (from the publication date noted on the first page of this article) for the purpose of providing a public and permanent scientific record, in accordance with Article 8.1 of the Code. The separate print-only edition is available on request from PLoS by sending a request to PLoS ONE, 1160 Battery Street, Suite 100, San Francisco, CA 94111, USA along with a check for $10 (to cover printing and postage) payable to “Public Library of Science”.

In addition, this published work and the nomenclatural acts it contains have been registered in ZooBank, the proposed online registration system for the ICZN. The ZooBank LSIDs (Life Science Identifiers) can be resolved and the associated information viewed through any standard web browser by appending the LSID to the prefix “http://zoobank.org/”. The LSID for this publication is: urn:lsid:zoobank.org:pub:90004C93-59CE-484B-949A-66B98EAC94B2.

Digital archives where PLoS articles are deposited are PubMedCentral (http://www.pubmedcentral.nih.gov/) and LOCKSS (http://www.lockss.org/).

## Results

### Molecular systematics

**Figure 3:**
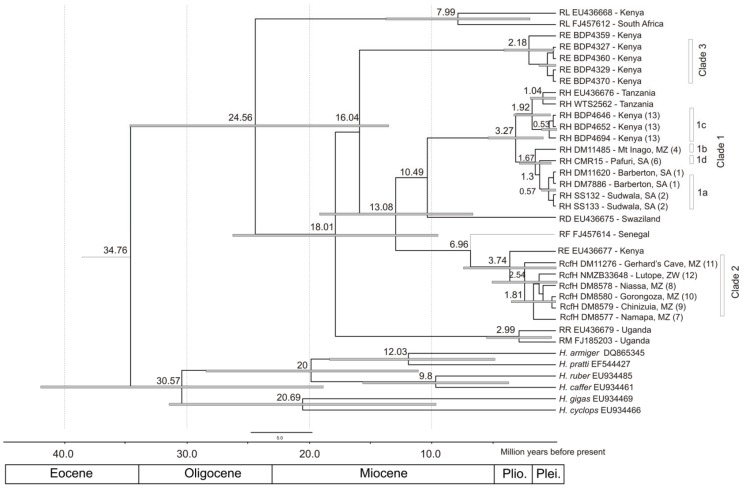
Consensus tree for the cytochrome b dataset for representative genotyped specimens of the *Rhinolophus hildebrandtii* complex. The topology represents the consensus topology from a 20 million MCMC run implemented in BEAST. Estimates of divergence times (million years ago; Mya) are indicated adjacent to nodes or above branches and grey bars indicate 95% HPD values. The split between the Hipposideridae and Rhinolophidae was used as the calibration point. Taxa names include museum/field numbers which correspond to Appendix S1 or GenBank accession numbers and abbreviations are: RcfH - *R. cf. hildebrandtii*, RD - *R. darlingi*, RE - *R. eloquens*, RF - *R. fumigatus*, RH - *R. hildebrandtii* s.l., RL - *R. landeri* and RR - *R. ruwenzorii*. Localities, where available, are provided, abbreviations include SA - South Africa, MZ - Mozambique, and ZW - Zimbabwe, and the numbers in parentheses correspond with place names in Table S1 and Fig. 2 for Clade 1 and 2 individuals.

Results from both parsimony and Bayesian analyses show similar topologies. For all four genes analysed, three well-supported, monophyletic clades are recovered: 1) Clade 1 comprising *R. hildebrandtii* from Mpumalanga Province in South Africa, Pafuri in the extreme northern region of Kruger National Park, Mts Mabu and Inago in Mozambique, Lutope-Ngolangola Confluence (Gorge) in Zimbabwe and Tanzania and Kenya (including topotypic samples of *R. hildebrandtii* from Taita District, Kenya); 2) Clade 2 comprising *R.* cf. *hildebrandtii* specimens from Mozambique and Zimbabwe, and 3) Clade 3 consisting of *R. eloquens* individuals from Kenya (Fig. 2; Fig. 3; Fig. S1).Within the *R. hildebrandtii* Clade 1, four lineages were recognized (1a, 1b, 1c and 1d/e based on the cytochrome b ultrametric tree in Fig. 3, and the 12S tree in Fig. S1 {1d and 1e are regarded on other characters to belong to the same species; see below}). These lineages correspond to broad or narrow geographical areas as follows: 1a) Sudwala Mine, Barberton and Mayo Mine, Mpumalanga Province of South Africa; 1b) Mts Mabu and Inago, northern Mozambique; 1c) Kenya and Tanzania; 1d/e) individuals from Pafuri, South Africa and the Lutope-Ngolangola Gorge, Zimbabwe (Fig. 3; Fig. S1).

Uncorrected pairwise sequence distances (p) for the four gene fragments are provided in Table 1, 2. Because of the high degree of variation in distances within clades and lineages, the values between individuals used in the control region dataset are provided in Table S2. On average, genetic distances between the three main clades were similar with the *R.* cf *hildebrandtii* individuals (Clade 2) being as different from *R. hildebrandtii* (Clade 1) as they were from *R. eloquens* (Clade 3). For cytochrome b, Clade 1 differs from Clades 2 and 3 by 7.7 to 9.3% and Clades 2 and 3 differ by *ca* 7%, whilst sub-lineages within Clade 1 varied from 0.6 to 1.1% (Table 1).

**Table 1:** ‘Uncorrected p’ pairwise sequence distances for the 12S gene below the diagonal and Chd1 gene above the diagonal for the clades (1, 2, 3) and some lineages (1a, 1b, 1c) identified in the molecular analyses of the *Rhinolophus hildebrandtii* complex.

	1a (*cohenae* sp. nov.)	1b (*mabuensis* sp. nov.	1c (*hildebrandtii* s.s.)	2 (*mossambicus* sp. nov.)	3 (*eloquens*)	Outgroup
**1a**	*0.000/* **0.000**	0.003	0.001	0.006	0.010	0.013
**1b**	0.005–0.011	∼/**0.003–0.011**	0.001	0.006	0.010	0.013
**1c**	0.008	0.011–0.016	*0.000/* **0.000**	0.005	0.009	0.012
**2**	0.022–0.027	0.022–0.030	0.025–0.030	*0.000/* **0.000–0.008**	0.006	0.009
**3**	0.027–0.030	0.033–0.038	0.025–0.027	0.027–0.035	*0.000*/**0.000–0.003**	0.013
**Outgroup**	0.030–0.033	0.038–0.041	0.030	0.033–0.041	0.033–0.036	*0.000*/**0.000**

Values in bold refer to within-clade distances for the 12S gene and values in italics refer to the within-clade distances for the Chd1 gene. Because of the high level of variation, no values are provided for the outgroup *Rhinolophus* and *Hipposideros* species for cytochrome b. Revised taxon names for molecular lineages are given in parentheses in the column headings (see Taxonomic Conclusions).

**Table 2:** ‘Uncorrected p’ pairwise sequence distances for the control region below the diagonal and cytochrome b gene above the diagonal for the clades (1, 2, 3) and some lineages (1a, 1b, 1c) identified in the molecular analyses of the *Rhinolophus hildebrandtii* complex.

	1a (*cohenae* sp. nov.)	1b (*mabuensis* sp. nov.	1c (*hildebrandtii* s.s.)	2 (*mossambicus* sp. nov.)	3 (*eloquens*)	Outgroup
**1a**	*0.000*/**0.000**	0.006	0.009–0.011	0.082	0.090–0.093	
**1b**	0.027–0.029	*∼*/**0.013**	0.009–0.011	0.077–0.080	0.088–0.090	
**1c**	0.032–0.040	0.029–0.036	*0.000–0.006*/**0.000–0.023**	0.077–0.080	0.088–0.090	
**2**	0.092–0.103	0.084–0.099	0.088–0.101	*0.000–0.009*/**0.000–0.023**	0.071–0.075	
**3**	0.082–0.094	0.073–0.086	0.073–0.086	0.080–0.096	*0.000–0.009*/**0.000–0.034**	
**Outgroup**	0.098–0.117	0.095–0.116	0.101–0.120	0.111–0.128	0.084–0.105	*∼/* **0.019–0.071**

Bold values indicate within-clade distance for control region and values in italics indicate within-clade distances for cytochrome b. Because of the high level of variation, no values are provided for the outgroup *Rhinolophus* and *Hipposideros* species for cytochrome b. Revised taxon names for molecular lineages are given in parentheses in the column headings (see Taxonomic Conclusions).

Bayesian divergence estimates suggest that divergence among the deeper lineages within the Rhinolophidae occurred during the Miocene. The most recent common ancestors for each of the three lineages representing *R. hildebrandtii* (Clade 1; *ca* 3.27 Mya), *R.* cf *hildebrandtii* (Clade 2; *ca* 2.54 Mya) and *R. eloquens* (Clade 3; *ca* 2.18 Mya) were present during the late Pliocene (Fig. 3). Within *R. hildebrandtii*, East African bats (Lineage 1c) diverged from Sudwala and surrounds (Lineage 1a) and Mt Inago (Lineage 1b) bats during the late Pliocene (*ca* 3.27 Mya; Fig. 3), with Lineages 1a and 1b diverging during the Pleistocene (*ca* 1.67 Mya; Fig. 3).

### Acoustic recordings and analysis

Data for peak echolocation frequency for different populations surveyed are summarised in Table 3. Estimates of peak frequency within *R. hildebrandtii* sensu lato varied from 32 to 46 kHz (Table 3). Where larger sample sizes were available, e.g., Sudwala and surrounds, Mpumalanga, South Africa (n = 7) and Lutope-Ngolangola Gorge, Zimbabwe (n = 15), standard deviations were very low indicating minimal intra-population variation. An obvious exception was the sympatric occurrence of two divergent sonotypes (37 and 46 kHz) at Lutope-Ngolangola Gorge (Table 3).

**Table 3:** Summary of data for peak echolocation frequency of *R. hildebrandtii* s.l. populations.

Locality	Revised taxon name	Clade/lineage	N	Mean frequency(kHz)	STDEV	Source
Kenya: several localities	*hildebrandtii* s.s.	1c	55	42.4	0.33	[15]
South Africa: Pafuri, Kruger National Park	*smithersi* sp. nov.	1d	?	40.0	-	[34]
South Africa: Pafuri, Kruger National Park	*smithersi* sp. nov.	1d	2	44.2	0.42	[30], This study
Zimbabwe: Lutope-Ngolangola	*smithersi* sp. nov.	1e	1	46.0	-	This study
South Africa: Mpumalanga Province (Sudwala Mine, Mayo and Barberton)	*cohenae* sp. nov.	1a	7	32.8	0.24	This study
Zimbabwe: Lutope-Ngolangola	*mossambicus* sp. nov.	2	15	37.1	0.28	This study
Mozambique: Namapa	*mossambicus* sp. nov.	2	1	38.5	-	This study
Mozambique: Chinizuia Forest	*mossambicus* sp. nov.	2	1	37.6	-	This study
Mozambique: Gorongoza Caves	*mossambicus* sp. nov.	2	1	34.9	-	This study
Mozambique: Gerhard's Cave	*mossambicus* sp. nov.	2	1	38		This study
Mozambique: Mt Mabu	*mabuensis* sp. nov.	1b	2	37.7	-	M. Curran & M. Kopp, personal communication

This integrates published and new data (this study). N indicates samples size; STDEV indicates standard deviation.

The revised names for clades are explained under Taxonomic Conclusions.

Of the five linear models of increasing complexity, the most complex model (Model 5) which included all ten variables, performed best (AIC 122.58), followed by Model 4 having eight variables (AIC 150.89), and Models 2 (AIC 161.79), 1 (AIC 163.50) and 3 (AIC 163.62) which all had two variables (CCL and altitude) but differed in whether altitude was allowed to interact with CCL (Model 1) or not (Model 2) and whether altitude was treated as continuous (Model 3) or categorical (all other models). Model 5 had residuals conforming closely to the normal distribution and explained 93% of variation in peak frequency of the CF component. Based on relative importance (using the “lmg” metric in R), two skull size parameters (CCL: 17.0%; NL: 19.0%) and altitude (17.5%) explained most of the variation, followed by forearm length (8.5%), relative humidity (6.2%), rostral chamber height (6.1%), zygomatic width (5.7%), rostral chamber width (5.6%), clade (5.2%), and sex (2.4%) (Fig. S3). CCL accounts for 68.3% of variation in peak frequency according to the regression equation: CF frequency = 142.59 - 4.29*CCL (Fig. S3a). Altitude (<600 m or >600 m) alone explains 35.3% of variation with populations from low elevations having significantly higher peak frequencies than high elevation populations (Fig. S3b). A positive and significant correlation also exists between altitude and CCL (R^2^ = 0.247; p<0.01; CCL = 23.73+0.0021*altitude); however we found no significant interaction between CCL and altitude (categorical variable) in our Model 2 (interaction β = −0.74; t = −0.51, p>0.05). When ten variables were considered simultaneously in a complex model, two cranial length variables (CCL and NL) and altitude had a relative importance (>17%) much greater than that for other variables (<10%). Forearm length (with relative importance of 8.5%) was significantly negatively correlated with peak frequency (R^2^ = 0.371; p<0.01; frequency = 101.17 - 1.006*FL), as was relative humidity (RH; relative importance 6.2%; R^2^ = 0.146, p = 0.02; frequency = 50.52 -0.245*RH).

### Morphology

#### Cranial morphometrics

**Figure 4:**
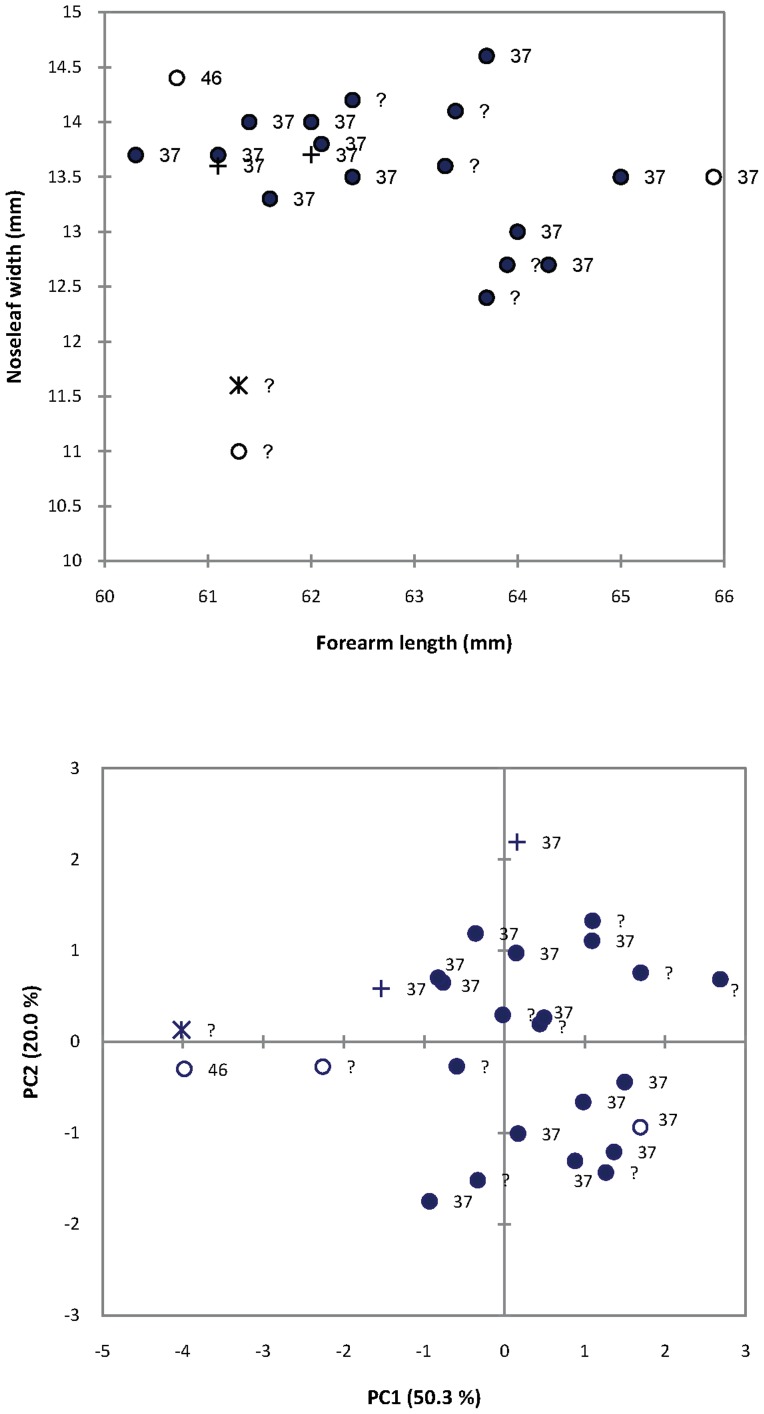
Morphometric variation in a series representing the *R. hildebrandtii* complex from Lutope-Ngolangola, Zimbabwe: a) biplot of forearm length versus noseleaf width and b) PCA of five craniometric variables (M3M3, CM3, IOC, NW, NH) in 26 individuals of known (37 or 46 kHz) and unknown (?) frequency. Females indicated by open circles, males by closed circles or crosses or asterisk. Voucher specimens for molecular sequencing study indicated by asterisk (Clade 1e:  = *smithersii* sp. nov.; see Taxonomic Conclusions) and crosses (Clade 2:  = *mossambicus* sp. nov.; see Taxonomic Conclusions). Hereafter, all individuals with a frequency of 37 kHz were assumed to belong to Clade 2 (*mossambicus* sp. nov.) and the 46 kHz individual was assumed to belong to Clade 1e (*smithersi* sp. nov.).

Firstly, we analysed external and cranial morphometric differences associated with two sympatric sonotypes (37 kHz and 46 kHz) from the Lutope-Ngolangola Gorge in NW Zimbabwe. In a sample of 14 individuals of known peak echolocation frequency for which we had complete morphometric data, a single female with peak echolocation frequency of 46 kHz displayed the second-lowest forearm length but the second-highest noseleaf width (disproportionately wide noseleaf comprising 23.7% of forearm length) compared to 13 individuals (one female and 12 males) recorded at 37 kHz (noseleaf comprised 19.7–22.9% of forearm length; Fig. 4a). The same 46 kHz individual was much smaller in cranial size (lower scores on PC1) than all other 37 kHz members of the population (Fig. 4b).

Of three individuals sequenced from this Lutope-Ngolangola sample, two have peak frequencies of 37 kHz (NMZB 33644 and NMZB 33648) and belong to Clade 2 whilst a third (NMZB 33652) belonged to Clade 1 (Fig. 3; Fig. S1). Although the echolocation call of the latter was not recorded in the field, it was noticeably small in cranial size and in fact grouped closely with the individual NMZB 33647 which had a peak frequency of 46 kHz (Fig. 4b). Albeit based on very small sample sizes, this correspondence between molecular, morphological and acoustic data suggests that the two acoustic sonotypes occurring sympatrically at Lutope-Ngolangola are also genetically and morphologically divergent, belonging to the two major clades defined by mitochondrial and nuclear sequences.

**Figure 5:**
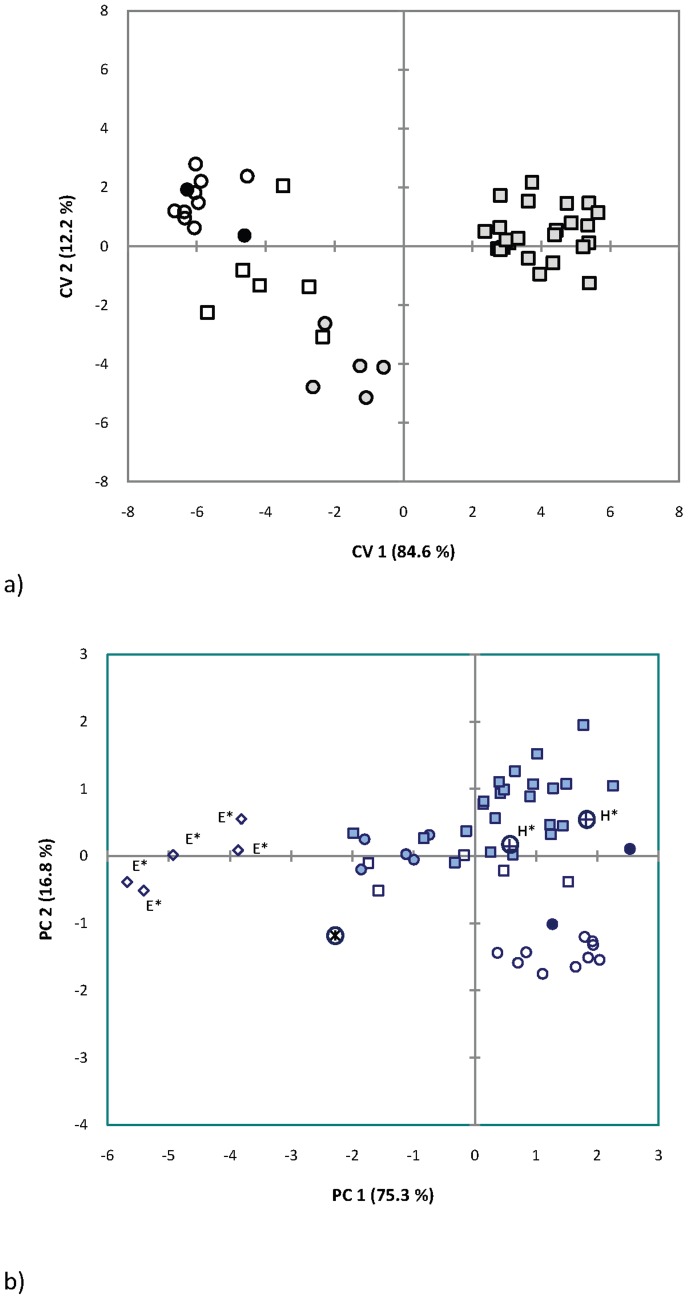
Canonical variates analysis (CVA) (a) of 10 cranial variables in five groups of the *Rhinolophus hildebrandtii* complex defined by molecular analysis; and PCA (b) of five cranial variables for sample in (a) with type series of *hildebrandtii* (“H*”) and *eloquens* (“E*”) added. Open circles = Clade 1a ( = *cohenae* sp. nov.); closed circles = Clade 1b ( = *mabuensis* sp. nov.); shaded circles = Clade 1d ( = *smithersi* sp. nov.; Pafuri); asterisk enclosed in circle = Clade 1e ( = *smithersi* sp. nov.; Zimbabwe); open squares = Clade 2 (*mossambicus* sp. nov.; Mozambique); shaded squares = Clade 2 (*mossambicus* sp. nov.; Lutope, Zimbabwe); open diamonds = *R. eloquens* type series (Clade 3); crosses in circles = *R. hildebrandtii* type and co-type (Clade 1c).

Secondly, using five cranial variables, we examined craniometric variation in a sample combining the above population with (1) recently collected voucher specimens from northern South Africa and Mozambique included in molecular and acoustic studies and (2) type series of *R. hildebrandtii* and *R. eloquens* from East Africa (Fig. 5b; Table 3). Based on PCA, the small-sized Clade 1 individual from Zimbabwe is clearly the smallest *R. hildebrandtii* s.l. individual from southern Africa (Fig. 5a; situated far to the left on PC1) but crania from the type series of *R. eloquens* from Entebbe, Uganda were much smaller than all other populations, including this small-sized *R. hildebrandtii*. The holotype and cotype of *R. hildebrandtii* fell within the range of variation of the population of 37 kHz *R. hildebrandtii* from Lutope-Ngolangola in Zimbabwe (Fig. 5a).

Members of Clade 1 displayed both extremes in cranial size relative to the moderately-sized Clade 2 sample. For example, whilst some members of Clade 1 (Lineage 1a from Sudwala and surrounds and Lineage 1b from Mts Mabu and Inago) could be clearly distinguished on the basis of their larger size (separation on PC1 and PC2 axes; Fig. 5b), individuals from Pafuri (Lineage 1d) and the small individual from Lutope-Ngolangola (Lineage 1e) were distinctly smaller in cranial size than most Clade 2 members of *R. hildebrandtii* from Mozambique lowlands and the 37 kHz individuals from Lutope-Ngolangola (which we also assigned to Clade 2; see above). Members of Clade 2 were intermediate-sized and varied considerably in skull size with some overlapping marginally with the Pafuri sample and others just bordering the lower end of the range of variation of the Sudwala sample (Fig. 5b). PCA indicated a clear-cut separation between the Sudwala sample and Mt Mabu and all other populations on PC2, with the former group having disproportionately narrow maxillae in relation to mastoid width (Fig. 5b, Table 3).

The trends mentioned above were confirmed by CVA of ten variables in five pre-defined groups (Fig. 5a) based on molecular clades and sub-clades (lineages) defined in Fig. 2 and Fig. S1: 1) Lineage 1a (Sudwala); 2) Lineage 1b (Mts Mabu and Inago); 3) Lineage 1d (Pafuri); 4) Clade 2 (Mozambique); 5) Clade 2 (Lutope-Ngolangola; 37 kHz individuals) (Fig. 5a). Based on CVA, 96% of individuals were assigned to their correct *a priori* group, all of Groups 1, 3 and 5 defined above but only one of two individuals of Group 2 and five of six individuals (83%) of Group 4 (Clade 2). Due to small sample sizes, Lineages 1c and 1e were not included in the CVA analysis.

**Figure 6:**
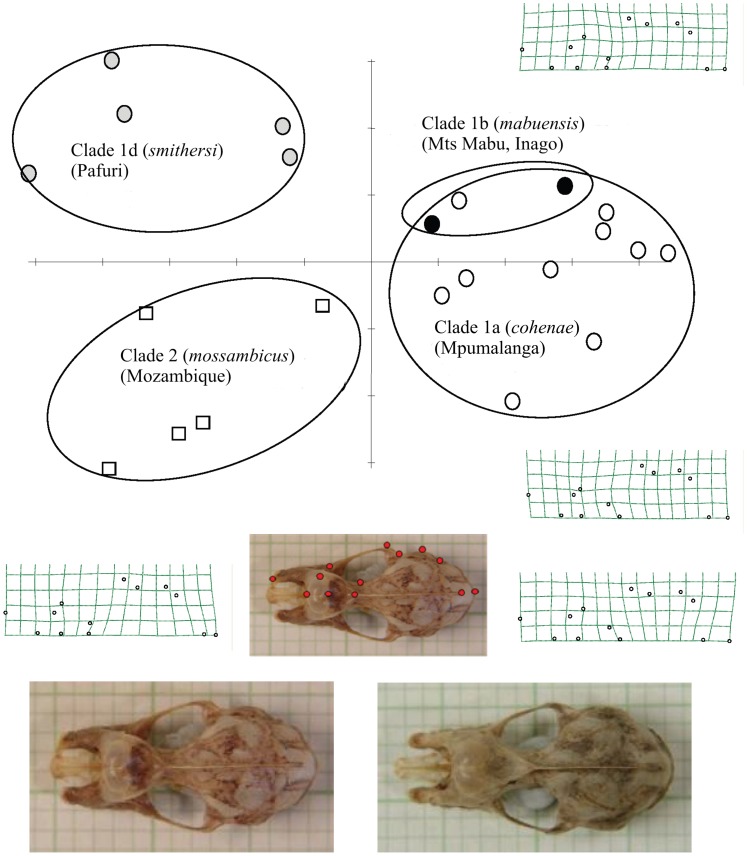
Relative warps analysis (RWA) of 13 dorsal cranial landmarks from 22 individuals of *R. hildebrandtii* s.l. belonging to two molecular clades and two lineages of Clade 1 (see **Fig. 3**). Revised taxon names are provided in parentheses (see Taxonomic Conclusions). Skulls which were included in this analysis are indicated in Table S1. Symbols as in Fig. 5. Thin plate splines (grids) show landmark distortions represented by extremes of variation on RW1 (left = negative; right = positive) and RW2 (bottom = negative; top = positive) axes. The two skull photographs at the bottom are of actual specimens representing the negative (left: TM 41997, *smithersi* from Pafuri) and positive (right: DM 11560, *cohenae* from Mayo, Mpumalanga Province) extremes of variation on RW1. Landmark positions (filled circles) are shown in the photograph in the centre.

**Figure 7:**
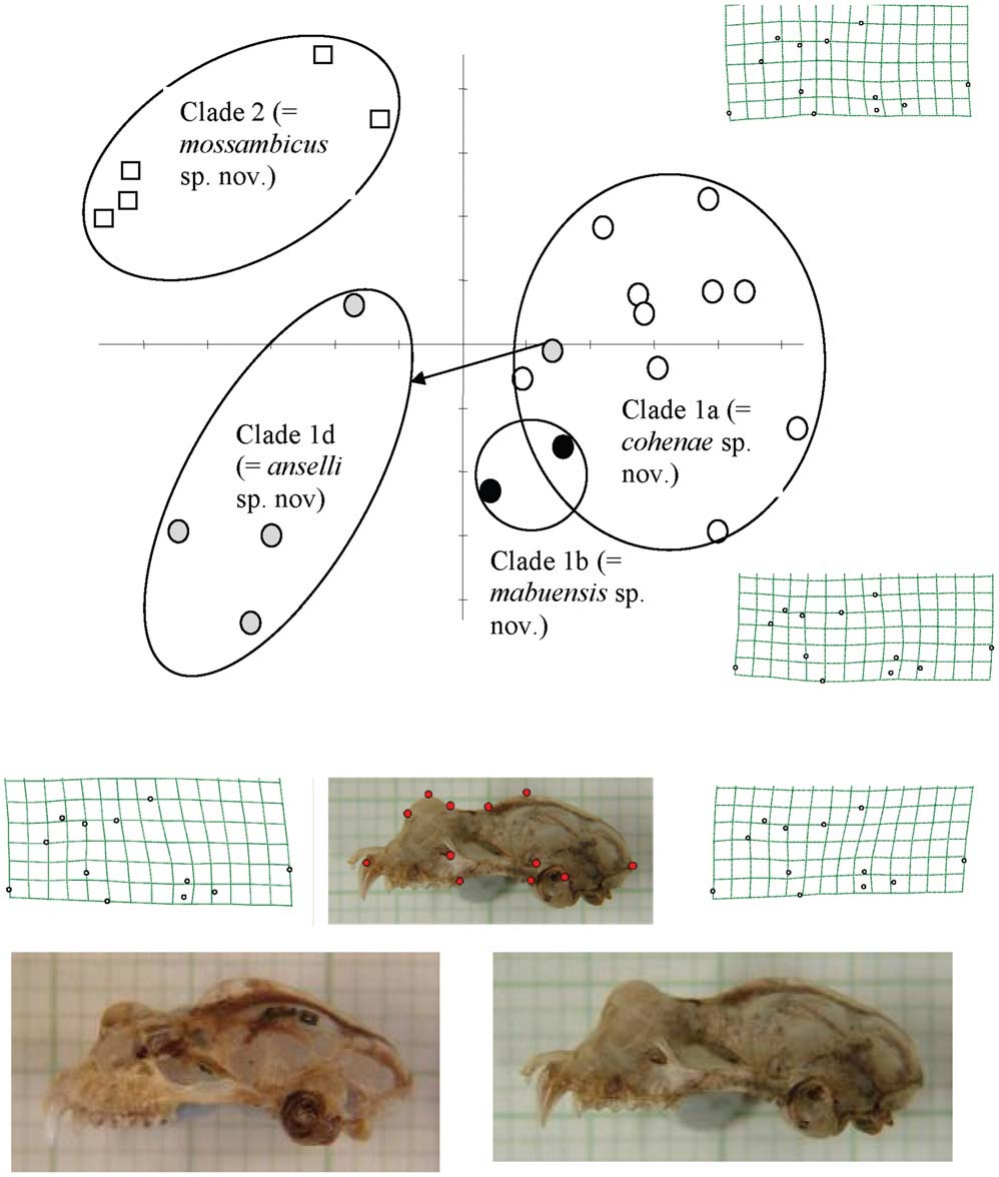
Relative warps analysis (RWA) of 12 lateral cranial landmarks from 23 individuals of *R. hildebrandtii* s.l. belonging to two molecular clades and two lineages of Clade 1 (see **Fig. 3**). Revised taxon names are provided in parentheses (see Taxonomic Conclusions). Skulls which were included in this analysis are indicated in Table S1. Symbols as is in Fig. 5. Thin plate splines (grids) show landmark distortions represented by extremes of variation on RW1 (left = negative; right = positive) and RW2 (bottom = negative; top = positive) axes. The two skull photographs at the bottom are of actual specimens representing the negative (left: DM 8577, *mossambicus* from Namapa, Mozambique) and positive (right: DM 11560, *cohenae* from Mayo, Mpumalanga Province) extremes of variation on RW1. Landmark positions (filled circles) are shown in the photograph in the centre.

To investigate possible subtle cranial shape differences between molecularly defined clades, the third step in our morphometric analysis was to analyse landmark data from dorsal (Fig. 6) and lateral (Fig. 7) cranial images. Relative warps analysis (RWA) of both dorsal and lateral landmarks distinguished the same three groups comprising: (1) Lineages 1a and 1b comprising populations from Sudwala and Mts Mabu and Inago; (2) the Pafuri population representing Lineage 1d; and (3) Clade 2 (lowland Mozambique) (Figs. 6, 7). In both dorsal and lateral analyses the first relative warp (RW1) distinguished the first group from the other two whilst RW2 distinguished groups 2 (Lineage 1d) and 3 (Clade 2). Thin plate splines (deformation grids) indicated shape differences associated with RW1 and RW2 (Figs. 6, 7). In dorsal view (Fig. 6), shape variation was associated with the relative position of the junction between the two supraorbital ridges and the sagittal crest, which shifted from anterior (Lineages 1d, 2) to posterior (Lineages 1a, b) resulting in a poorly- to well-developed V-shaped basin (“frontal depression” [12]) respectively behind the nasal inflation. Another cranial difference relates to the length of the sagittal crest (shorter in Pafuri and lowland Mozambique; longer in Sudwala, Mt Mabu and Mt Inago). These two above-mentioned characters (frontal depression and sagittal crest) are linked because they share a point. Based on the thin plate spline for RW2 in Fig. 6, Pafuri (Lineage 1d) was distinguished from lowland Mozambique (Clade 2) by having a partially developed frontal depression (intermediate condition). In lateral view (Fig. 7), the main differences (along RW1) related to the development (and relative height) of the nasal inflation in relation to the sagittal crest. In individuals from Pafuri and lowland Mozambique the sagittal crest was prominently developed and clearly rises above the nasal inflation in lateral view whilst in Sudwala, Mt Mabu and Mt Inago the sagittal crest was noticeably less developed and did not rise appreciably above the line of the well developed nasal inflation. Based on RW2 in Fig. 7, Pafuri was distinguished from lowland Mozambique in having a noticeably smaller nasal inflation in lateral view and a generally flatter lateral profile.

Fourthly, we used PCA to analyse a large sample derived from museum collections in relation to the above-mentioned voucher specimens (Fig. S2). Due to significant sexual dimorphism detected by provisional analyses, we limited our analysis to 109 males of *R. hildebrandtii* s.l. and *R. eloquens* s.l. from seven countries throughout the range of both species. Apart from Clade 1a (from Sudwala) which separates from all other populations on its larger size, and type series of *R. eloquens* which are mostly separated from *R. hildebrandtii* s.l., all other OTUs of *R. hildebrandtii* overlap considerably. There is a tendency for northern Zimbabwe individuals to plot above those from the remainder of Zimbabwe on PC2, and for specimens from Zambia, Malawi and East Africa to plot within the northern Zimbabwe group. Two males from lowland Mozambique belonging to Clade 2 are slightly larger in size (higher PC 1 scores) and group just to the right of the northern Zimbabwe group. Thus, morphometric analysis at this continental scale is uninformative, because it cannot diagnose nor delimit the clades revealed by molecular data.

#### Noseleaf

**Figure 8:**
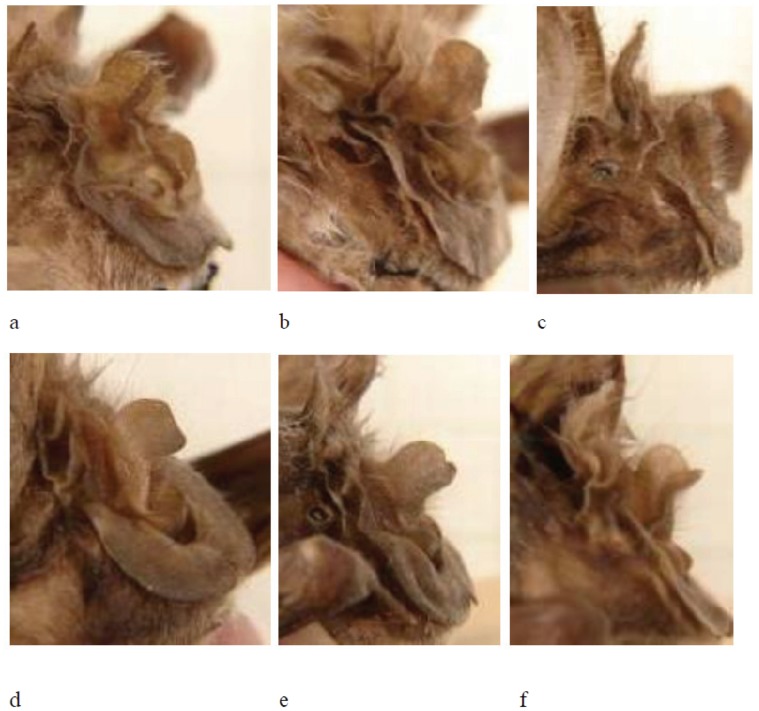
Photographs showing lateral views of noseleafs of selected individuals (including holotypes of new species) of the *Rhinolophus hildebrandtii* complex representing molecular Lineage 1a ( = *cohenae* sp. nov.; a–b), Lineage 1b ( = *mabuensis* sp. nov.; c) and Clade 2 ( = *mossambicus* sp. nov.; d–f). a = DM 7886 (*cohenae* sp. nov.;Barberton Tunnel, Mpumalanga Province, South Africa); b = DM 8626 (*cohenae* sp. nov.; Barberton Tunnel, Mpumalanga Province, South Africa; Holotype); c = DM 10842 (*mabuensis* sp. nov.; Mt Mabu, Mozambique; Holotype ); d = DM 8578 (*mossambicus* sp. nov.; Niassa Game Reserve, Mozambique; Holotype); e = DM 8579 (*mossambicus* sp. nov.; Chinizuia, Mozambique); f = DM 8577 (*mossambicus* sp. nov.; Namapa, Mozambique).

Considerable variation exists in noseleaf shape as exemplified by representative lateral photographs of six individuals shown in Fig. 8. While the more typical low, rounded shape [12] is apparent to some degree in Figs. 8a, b, d, e, the specimen from Mt Mabu (Fig. 8c) had a very distinctive and atypical shape comprising a small rounded bump. Another specimen from Namapa, Mozambique (Fig. 8f) also had an atypical shape in lateral profile (more high and rounded). Our sample of noseleafs is limited to material collected from live animals during this study, so it does not represent all lineages; however, our aim was to demonstrate the variability of this character even within lineages, hence its unreliability for characterising the molecular clades recognised by this study. Such variability has also been detected based on studies of very large series of museum specimens of this species (FPDC, unpublished data).

#### Baculum

**Figure 9:**
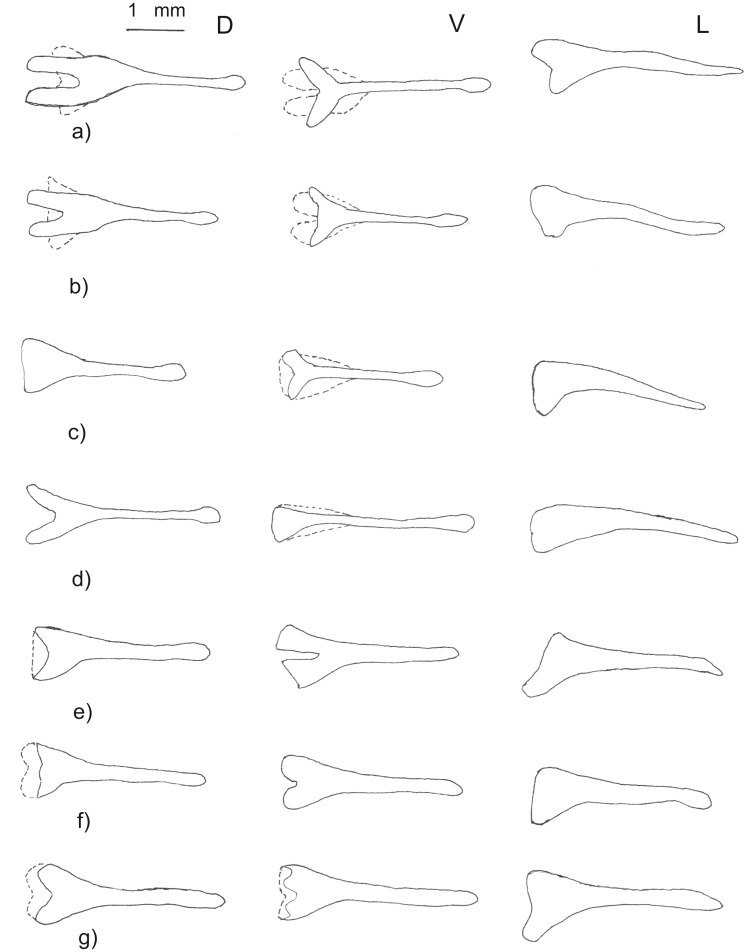
Dorsal (D), ventral (V) and lateral (L) view of bacula (tips on right) from four individuals (a–d) from Mpumalanga (Clade 1a = *cohenae* sp. nov.), two (e–f) from lowland sites in Mozambique (Clade 2 = *mossambicus* sp. nov.) and one (g) from Mt Mabu in Mozambique (Clade 1b = *mabuensis* sp. nov.). a = DM 11558 (Sudwala); b = DM 11620 (Barberton Tunnel; Topotype of *cohenae*); c = DM 11560 (Mayo); d = DM 11618 (Barberton Tunnel); e = DM 8580 (Gorongosa); f = DM 8578 (Niassa GR; Holotype of *mossambicus*); g = DM 10842 (Mt Mabu; Holotype of *mabuensis*). Bacula of Clade 1a (*cohenae* sp. nov.) have spatulate tip (rounded in Clades 2 (*mossambicus* sp. nov.) and 1b (*mabuensis* sp. nov.)), typically emarginated basal portion (less so in Clades 2 and 1b) and shaft laterally compressed (cylindrical in Clades 2 and 1b) and sloping downwards in lateral view (horizontal in Clades 2 and 1b).

All bacula observed were symmetrical (in dorsal and ventral views) and buccinate (trumpet-shaped) with a long, narrow, slightly laterally-compressed shaft and a well-developed bulbous basal portion (Fig. 9). Examination of a series of intact bacula from eight males collected from three closely spaced roosts from Sudwala and surrounds (Lineage 1a) revealed subtle intra-population variation (Fig. 9a–d). Total length varied from 2.76 to 3.64 mm (n = 8; mean = 3.39 mm; s.d. = 0.28). The basal portion was typically deeply emarginated in dorsal view (with the exception of DM 11560 where it was not emarginated), with the ventral view showing variable degrees of emargination from not emarginated (DM 11620) to conspicuously emarginated (DM 11558). The degree of development of the ventral and dorsal emarginations determined the shape in lateral view. In five bacula with well-developed emarginations, the height of emargination ranged from 0.72 to 0.97 mm (mean = 0.82; s.d. = 0.10), representing approximately 24% of the total length of the baculum.

Bacula from two males from molecular Clade 2 (Niassa and Gorongoza in Mozambique) were available for comparison with the Clade 1a sample. In size and shape they varied slightly but significantly from those from Clade 1a; total length of the two Clade 2 bacula varied from 3.14 to 3.30 mm. The shaft was horizontal with no downwards inflection, cylindrical rather than compressed laterally; the shaft tip was a rounded point rather than slightly spatulate and the bulbous portion was only slightly emarginated in dorsal and ventral views (Fig. 9e, f). We refer hereafter to the bacular shape represented by Clade 1a as “Type 1” and the shape represented by Clade 2 as “Type 2”. The bacula of the Mt Mabu animal (Clade 1b) is similar in size to Type 1 (3.4 mm) but in shape clearly conforms to Type 2 in its horizontal and cylindrical shaft, weakly-emarginated distal portion and rounded tip (Fig. 9g).

Drawings (by FPDC) of dorsal and lateral views of bacula from a series of 13 *R. hildebrandtii* s.l. males from Marondera, eastern Zimbabwe (NMZB 62133–62139; 62141–62142; 62144–621477) and one from Esigodini, central Zimbabwe (NMZB 80237; total length 4.2 mm) revealed a structure most similar to lowland Mozambique specimens (Type 2), with little or no emargination of the bulbous portion. In contrast, an individual from the Luangwa Valley in Zambia (NMZB 62149; total length 2.6 mm) had a structure with marked emargination similar to those in the Mpumalanga sample (Fig. S4).

Our sample for analysis of bacular morphology was limited largely by availability of male specimens of different lineages freshly collected during this study (only Lineages 1a, 1b and 2). Thus Lineages 1c-e are not represented. The sole specimen recorded for Lineage 1e was a female. For logistic reasons it was not possible within the time frame of this study to access bacula from loans of male specimens from various museum collections representing Lineages 1c and 1d. Future studies should seek to obtain a complete coverage of bacular descriptions for all lineages by loaning intact male specimens from historical museum collections, augmented by new genotypyed specimens.

## Discussion

### Molecular systematics

Our study aimed to resolve the species composition of the African *R. hildebrandtii* complex using a synthesis of multi-disciplinary evidence. It has been motivated by the initial discovery of distinct sonotypes in southern Africa within *R. hildebrandtii*, an apparent monotypic species. Our integrated analyses of these combined datasets not only reveal unprecedented diversity, previously overlooked in this species complex, but remarkably complex biogeographical structuring of hitherto unrecognized lineages. The molecular genetic data provide the overarching structure, which frames interpretations of regional biogeographical patterns of variation in morphology and sonar calls.

Analyses of three mitochondrial and one nuclear gene consistently recovered three reciprocally-monophyletic clades (Fig. 2, Fig. S1) corresponding to the species *R. hildebrandtii* s.s. (Clade 1), *R*. cf. *hildebrandtii* (Clade 2) and *R. eloquens* (Clade3). Craniometric data and morphological analyses which included measurements from the type series for *R. eloquens* and *R. hildebrandtii* confirm that individuals used in this study that correspond to Clade 2 are not *R. eloquens*, the horseshoe bat species most similar morphologically to *R. hildebrandtii*. In the cytochrome b analysis, bats from Clade 2 group with a sequence downloaded from GenBank (EU436677) which is labelled as *R. eloquens*, but does not group with the *R. eloquens* Clade 3.Verifiable credentials to recheck the identification of this specimen linked to the GenBank sequence are not available, a problem not unknown with GenBank-derived sequences. On the other hand, specimens in Fig. 2 and Appendix S1 referred to *R. hildebrandtii* s.s. (from Taita-Taveta and Makuendi Districts, Kenya) and *R. eloquens* (Malindi District, Kenya) were collected and identified with a high degree of confidence by Dr Bruce Patterson, a reputable bat taxonomist at the Field Museum of Natural History.

*Rhinolophus hildebrandtii* (Clade 1) is characterized by high levels of craniometric variation and divergent sonotype groups. Molecular analyses recovered three lineages with strong geographic structuring (however, only Lineage 1a, Sudwala and surrounds, is supported by the nuclear marker) and a possible fourth and fifth lineage for Pafuri (Lineage 1d) and Lutope-Ngolangola (Lineage 1e) respectively (Fig. 2, Fig. S1b).

### Morphological and acoustic divergence

Divergence in echolocation call frequency among species of horseshoe bats has been attributed to a number of determinants including allometry [54], [55], [56], ecomorphological constraints (e.g. dietary specialisation [17], [57]), circumventing moth hearing in prey detection [58], [59], facilitation of communication between conspecifics [17], [20], [60], [61], sexual dimorphism and ontogenetic variation [62], foraging habitat [56], [63], genetic drift [64] and environmental factors such as relative humidity which can enhance atmospheric attenuation of high frequency sounds [60]. Whilst variation of echolocation (pertinently peak frequency) within horseshoe bats is correlated with body size [56], [61] this relationship was not found within some species [33]. Moreover, during evolutionary history, shifts in echolocation frequency may have occurred before body size changed in lineages. This suggests selection may act directly on echolocation frequency due to other selection pressures, for example the need for reliable recognition and communication amongst conspecifics [56], [61].

Although a substantial body of evidence supports the relationship between body size and frequency in bats (including the horseshoe bats), a closer relationship may exist between frequency, and the attributes of the aural and auditory organs than with overall body size [65]. Significant relationships are similarly demonstrated between call frequency and noseleaf width [66], skull length and associated cranial characters [33]. Here, we show that skull size (and to a lesser extent body size as measured by forearm length) is inversely and significantly correlated with peak frequency within the *R. hildebrandtii* s.l. complex in concordance with the allometry hypothesis (Fig. S3). Most of the variation in peak frequency can be explained by differences in CCL (68.3%). However, other variables including noseleaf width, forearm length, relative humidity, and altitude may also contribute to divergence of echolocation call frequencies. Altitude and CCL are significantly correlated and altitude may influence CCL through Bergman's Rule whereby higher-altitude populations have larger body size. Thus we cannot exclude the possibility of altitude exerting an indirect negative influence on peak frequency through its positive correlation with skull size.

Although character divergence could reflect the outcome of drift in vicariant populations, drift alone does not adequately explain the origins of these dwarf and giants in divergent *Rhinolophus* clades. Morphometric analyses in this study reveal extreme divergence in cranial size and shape among the lineages comprising Clade 1, with giants occurring in habitat islands at higher-elevation (>600 m) on Mts Mabu and Inago (northern Mozambique), and the northern Drakensberg Range (Mpumalanga, South Africa), and dwarfs represented by the low-lying Pafuri population in the Limpopo Valley of northeastern South Africa (300 m) and the 46 kHz individual from the Lutope-Ngolangola Confluence in Zimbabwe (1000 m) which occurs sympatrically with larger-bodied 37 kHz individuals. Given the proximity of Lutope-Ngolangola to the lower-elevation Zambezi Valley, and the association between altitude, skull size and peak frequency, it is possible that the small-sized 46 kHz population (Lineage 1e) speciated within the lower-lying Zambezi Valley, and has subsequently expanded its range marginally onto the Zambezi Escarpment.

### Biogeographical Reconstruction

Our molecular dates constrain founding divergence events within the Rhinolophidae to the Oligocene and early Miocene (Fig. 2). The origin of these deeper lineages is consistent with previous studies [56], [67], which constrain the Old World radiation of the Rhinolophidae into their various species-groups during the mid-Cenozoic. It is noteworthy that the main lineages in other chiropteran radiations are also constrained as early Miocene (e.g. *Myotis* [68]; Phyllostomidae [69]). Global cooling that began in the middle Miocene [70] increased aridity across Africa, with subsequent contraction of forests, and the expansion of savanna woodlands and grasslands [71], [72], [73], [74].

Furthermore, a persuasive body of evidence testifies to late Miocene uplift of Africa, with its pronounced focus elevating the south-central and eastern regions [75], [76], [77], [78], [79], [80]. This represents the Plio-Pleistocene extension of the East African Rift System across southern and central Africa [79]. Thus the interacting determinants of uplift and climate accentuated topographic relief across the Afromontane Archipelago through the late Neogene [75], [78]. This paleo-climatic and tectonic forcing is invoked as the ultimate cause of allopatric speciation in these African horseshoe bats.

The East African lineages of the *R. hildebrandtii* complex diverged from the southern African bats during the late Pliocene (*ca* 3.27 Mya), whilst divergence within the southern African bats (between Lineages 1a, 1b and the Pafuri and Lutope-Ngolangola individuals) occurred during the Pleistocene (Figure 2). Individuals in Lineage 1b (Mts Mabu and Inago) diverged in the late Neogene (Plio-Pleistocene), when seasonal woodland/forest habitats were being replaced by more open woodland [81] and may have become isolated from other low-lying populations. Similarly, the lineage leading to 1a (Sudwala-Mpumulanga) and 1c (east African individuals) diverged at the beginning of the Pliocene, and populations may have been isolated due to changes in habitat. Climate driven forest-dynamics during this time period can be invoked to have driven species-level diversification in both forest and savanna adapted species. Indeed, Pliocene dynamics of African forests have been a primary driver of African bird speciation, with significant events across the Eastern Arc Mountains [82], [83].

Studies in a chronobiogeographical context [84], [85], with a focus on east African biodiversity, include herpetofauna [86], [87], birds [82] and mammals [88], [89], [90], [91], [92] including *Rhinolophus* [this study]. Congruence in their signatures support a late Neogene pulse of diversification across the Afromontane Archipelago [10], [11], which further concurs with botanical evidence [93].

Differentiation of plant and animal communities occupying scarp and montane forests along the southeastern escarpment of South Africa has been attributed to habitat fragmentation, during glacial maxima, when vicariance of subtropical forests isolated ancestral populations [94]. These palaeoclimatic dynamics also appear to explain lineage diversification patterns in the molossid bat, *Chaerephon pumilus* [95]. In addition to the tectonic forcing mentioned above, similar paleo-environmental forcing linked to older (Plio-Pleistocene) glacial maxima could explain cladogenesis in these *Rhinolophus*. Vicariance of forest habitats into climatically distinct ancestral refugia explains the enigmatic sister relationship between the dwarfs (Lineages 1d, Pafuri, and 1e, Zimbabwe) and giants (Lineage 1b, Mts Mabu and Inago, northern Mozambique). We propose that the postulated origin of these lineages during the late Pliocene and Early Pleistocene (Fig. 2) resulted in dwarfism in isolated relic (island) populations trapped in lower-elevation refugia in the Limpopo and Zambezi Valleys, and gigantism in island populations presumably trapped on higher-elevation escarpments and mountains. It is possible that coastal, low-lying populations became extinct during glacial maxima. A remarkably similar scenario invokes the upper reaches of major Malagasy rivers serving as retreat-dispersion corridors during glacial maxima when extinction occurs along lower reaches. This process may explain the biogeographic evolution of centres of microendemism in Madagascar's fauna [96].

With respect to inter-glacials, we invoke the earlier suggestion [94] that tropical forest species expanded their ranges southwards, pertinently in the prolonged mesic, warmer conditions of Marine Isotope Stage (MIS) 11 in the Mid-Pleistocene [97], [98]. This mechanism accounts for range expansion by the founding populations of Clade 2. After divergence from their sister lineage (represented today by *R. fumigatus* in East Africa during the late Neogene), Clade 2 lineages expanded southwards and westwards through lowland savanna habitats to establish secondary contact with Clade 1 (as evident at Lutope-Ngolangola Gorge on the Zambezi Escarpment). Although *R.* cf *fumigatus* (genotyped from Senegal) represents the sister clade to Clade 2, the phylogenetic affinities of the *R. fumigatus* complex remain unclear [13] and only further research can establish affinities among representatives of this species complex in eastern and southern Africa. We further note that the published genotype attributed to *R. fumigatus* from Tanzania [56] (Genbank Accession number for cytochrome b: FJ185197) (WTS 1533) actually represents *R. deckenii* (F.P.D. Cotterill *unpublished data*).

In summary, geomorphic and climatic evolution events across the Afromontane archipelago of eastern and southern Africa has had significant impacts on the evolution of these horseshoe bats. We hypothesize that Clades 1, 2 and 3 diverged in the early Miocene in response to tectonism forging the East African Rift Valley and associated relief, and regional climate-forcing; Clade 1 (*hildebrandtii* s.l.) became associated with montane forests, whilst Clade 2 (*mossambicus*) radiated into lower-lying savannas extending into SE Africa. Clade 3 (*eloquens*) has remained restricted to the forested highlands of the Eastern Arc and East African Rift. We postulate that tectonism and/or climate-forcing fragmented the widespread forests of the Miocene, isolating populations within Clade 1.

### An allometric speciation hypothesis

We suggest that speciation in these *Rhinolophus* involved an interesting mechanism of allometrically linked character divergence, which evolved as an effect of allopatry caused by paleo-environmental changes. It can be argued that genetic drift was important in vicariants (smaller effective population size), and selection also acted on dichopatric isolates in Afromontane habitats. Here, lower ambient temperature at higher altitudes selected for larger body size in Afromontane populations (invoking Bergman's Rule) to result in pronounced divergence in skull size. Significantly, the decrease in peak frequency of sonar calls of these Afromontane horseshoe bats constitutes allometric character divergence – the effect of increased skull length. Thus divergence in the CF sonar system was an indirect result of ecophysiological adaptation. The evolutionary consequences are represented in populations distributed across major river valleys (Zambezi and Limpopo), where *R. smithersi* sp. nov. is characterized by small body size (“dwarfism”) and higher echolocation frequency. In contrast, populations confined at higher elevations evolved larger size (“giants”) and lower echolocation frequencies (*R. mabuensis* sp. nov. restricted to mountains in northern Mozambique, and *R. cohenae* sp. nov. on the northern Great Escarpment of South Africa).

In summary, this Allometric Speciation Hypothesis attributes speciation in this species-complex of *Rhinolophus* to divergence in CF calls associated with adaptive shifts of body size in ancestral populations, which responded directly to Neogene habitat vicariance across the Afromontane Archipelago. Our evidence endorses Cracraft's argument that speciation is an effect of lower-level processes [99], because allometrically linked characters (notably sonar call structure) evolved indirectly to ecophysiological adaptation, pertinently larger body size. The mode of speciation in these horseshoe bats, in allopatry, was incidental to organismal adaptation [41], [100], shaped by the causal cascade initiated by palaeoenvironmental forcing. Pertinently, divergence in CF sonar-calls in respective lineages explains how Specific-Mate-Recognition Systems (SMRS) evolved in allopatry [41], [100], [101]. It is noteworthy that these SMRS have subsequently maintained lineage cohesion where geographical ranges of lineages expanded into sympatry.

In light of recent interest in understanding how the tempo and mode of macroevolution has shaped speciose clades [101], [102], [103], [104], [105], this “Allometric Speciation Hypothesis” explains the interplay between historical causes and effects. In *Rhinolophus*, peak echolocation frequency is of focal evolutionary relevance amongst the organismal traits comprising the CF sonar complex, because repeated shifts in this trait can explain why species richness has increased in this chiropteran clade. This mechanism points to a propensity for palaeoenvironmental vicariance to elevate speciation rates in *Rhinolophus* (and CF bats in general). The Allometric Speciation Hypothesis, proposed here, has interesting implications for the ‘harmonic-hopping’ [17] invoked to explain CF call divergence of *Rhinolophus* [17], [20] and *Hipposideros* [18], [19] across southeast Asia. Here their vicariant distributions [mapped in 12], [106] point to ultimate control by the tectonism that radically reshaped landmasses across this region [107], [108], [109].

This Allometric Speciation Hypothesis can be tested by: 1) identifying the molecular determinants (pertinently in transcriptomes) differentiating the species-specific behavioural and morphological traits comprising the CF sonar system, 2) behavioural-response experiments on CF bats, and 3) comparing the tempo and mode of macroevolutionary trends in FM versus CF bats. The latter should reveal higher rates of lineage diversification as an effect of SMRS evolution in allopatry.

### Taxonomic conclusions

Table 5 summarizes diagnostic traits and biogeographical features of five lineages within *hildebrandtii* s.l. diagnosed in this study. These lineages are recognised under the Evolutionary Species Concept as recognisably distinct species, as operationalized by the Phylogenetic Species Concept and additionally the Recognition Species Concept [41], [100], [110], [111]. We do not advocate the Genetic Species Concept [112] which would only recognize Clades 1, 2, and 3 (not sub-clades of Clade 1) due to arbitrary thresholds of genetic distance (*ca* 5% cytochrome b divergence). Recent studies of Afro-Malagasy molossid bats have shown that “good” morphological species occur in sympatry with congeneric species, yet exhibit cytochrome b divergences of as little as 1.3% (*Chaerephon pusillus*) to 2.3% (*C. leucogaster*) from respective congeners [113]. Further we reject the notion of subspecies to be an arbitrary construct associated with the Biological Species Concept (see [41] for criticism of the polytypic Biological Species Concept as applied to rhinolophid taxonomy). Thus, we regard as good species those allopatrically isolated lineages with diagnosable differences in characters, including characters likely selected for mate recognition and/or reproductive compatibility. Such pertinent characters include distinct sonar calls and bacula morphology.

### *Rhinolophus cohenae* new species

urn:lsid:zoobank.org:act:D0E1C9DD-7D36-4C01-AE0D-60AD474960A0

Cohen's Horseshoe Bat

Fig. 4, 4, Fig. S1, Table 3, 4, Table S1, Appendix S1

**Table 4:** Eigenvectors for PCA of recently collected voucher specimens of the *Rhinolophus hildebrandtii* complex associated with molecular and acoustic analyses, which includes type specimens of *R. hildebrandtii* and *R. eloquens* (see Fig. 5a).

	PC1	PC2	PC3
CCL	0.492	−0.119	−0.356
ZW	0.472	0.041	0.762
MW	0.445	−0.486	0.162
M3M3	0.309	0.865	−0.009
C1M3	0.491	−0.025	−0.517

#### Holotype

DM 8626; adult male, alcohol skin, skull and prepared baculum (Fig. 9f), collected by L. Cohen, 28 September 2004. Skull and mandible in good condition.

#### Type locality

Barberton, Mountainland Nature Reserve, 68 km SE Sudwala, Mpumalanga Province, South Africa, 25° 43′ 8″S; 31° 15′ 58″ E; elevation 690 m asl.

#### Diagnosis

Lowest recorded peak frequency (33.0 kHz in the holotype; mean 32.8±0.24 kHz, n = 7 in type series) which immediately distinguishes this species from all others. Noseleaf extremely wide, 13.5–16.3 mm (15.5 mm in holotype), exceeding the previously described range (12–15) for the species [12]. Body size and cranial size very large (Table 5; FL 66–68 mm; holotype 65.9 mm; GSL 29–30 mm; CCL 24–26 mm; holotype 29.9 mm and 25.6 mm respectively). In body and cranial size this species overlaps with *mabuensis*; however the latter species has a distinct echolocation frequency (*ca* 38 kHz) and a distinct baculum (“Type 2”). Anterior upper premolar conspicuous and located within tooth row or entirely absent. In the holotype and in three additional specimens, the anterior premolar is prominent and located within the toothrow. In another three specimens, the anterior premolar is absent (and the canine and posterior premolar in contact). In another specimen the prominent anterior premolar is situated in the toothrow on the right side but absent on the left side, leaving a small gap between canine and posterior premolar. In no cases were specimens found to have small anterior premolars situated external to the toothrow, thus distinguishing this species from *hildebrandtii* s.s. [12]. The baculum (total length 3.5 mm in holotype) has a unique shape (“Type 1”; Fig. 9) characterised by narrow, laterally compressed and ventrally deflected shaft, emarginated distal portion and spatulate tip, which is not found in other species of the *hildebrandtii* complex in which the baculum has been described (*mossambicus* and *mabuensis*). Genotypes of *R. cohenae* are members of Clade 1a (Fig. 2).

**Table 5:** Matrix showing diagnostic traits and distribution of seven evolutionary lineages within the *Rhinolophus hildebrandtii* complex.

Clade	Species name (& holotype number)	Distribution	Freq. (kHz)	NLW	FL	GSL	CCL	Relative height of rostral chamber and sagittal crest in lateral view (Fig. 6)	Depth of cranial profile overall (Fig. 6)	Frontal depression in dorsal view (Fig. 6)	Baculum (Fig. 8)	Relative size and position of anterior upper premolar
1a	*cohenae* sp. nov	Mpumalanga Province, South Africa	33	15.3; 13.5–16.3 (7)	66.4; 66–68 (10)	29.8; 29–30 (10)	25.3; 24–26 (10)	Rostral chamber> = sagittal crest	Flatter	Conspicuous& deep	Type 1	Prominent, in toothrow (or absent)
	(DM 8626)	Mountainland Nature Reserve, Barberton	33	15.5	65.9	29.9	25.6	As above	As above	As above	Type 1	Prominent in toothrow
1b	*mabuensis* sp. nov.	Mts Mabu & Inago, N Mozambique	38	15.0; 15.0–15.1(2)	67.5; 66–69 (2)	29.7–30.3 (2)	25.6; 25–26 (2)	Rostral chamber > = sagittal crest	Flatter	Conspicuous & deep	Type 2	Prominent - small, internal-external
	(DM 10842)	Mt Mabu	*ca* 38*	15.0	66.1	29.7	25.2	As above	As above	As above	Type 2	Prominent, partially in toothrow
1c	*hildebrandtii*	East Africa (Kenya, Tanzania)	42	11.0; 9.8–13.1 (9)	62.5 60–68 (8)	28.0 27–29 (4)	24.0 23–25 (6)	-	-	Shallow to moderate [12]	-	Moderate - small, external
	(ZMB 5378)	Ndi, Taita, Kenya	Ca 42 **	9.8	63.5	27.6	-	-	-	-	-	-
1d/e	*smithersi* sp. nov.	Pafuri, South Africa & Lutope-Ngolangola Gorge	44–46	11.3;10.1–14.4 (6)	62.6; 61–64 (5)	27.3; 27–28 (4)	23.5 23–24 (6)	Sagittal crest>rostral chamber	Intermediate	Weakly developed	-	Small, external
	(NMBZ 33647)	Lutope-Ngolangola Gorge, Zimbabwe	46	14.4	60.7	26.5	23.1	-	-	-	-	-
2	*mossambicus* sp. nov.	Mozambique, extending to NW Zimbabwe	35–38	13.5; 12.9–14.2(3)	62.8; 60–65 (5)	28.1; 27–29 (5)	24.4; 24–25 (5)	Sagittal crest≫rostral chamber	Higher & domed	Reduced	Type 2	Minute, external
	(DM 8578)	Niassa Game Reserve, N Mozambique	*ca* 35–38 ***	14.2	63.9	28.5	25.0	As above	As above	As above	As above	As above
3	*eloquens*	East Africa	-	9.7;9.4–10.6(4)	55.5; (54–57(4)	25.2;25.1–25.3(3)	22.0;21.7–22.2(5)	-	-	Shallow[12]	-	Minute, external
	(BM 1899.8.4.4)	Entebbe, Uganda	-	10.6	56.1	25.1	21.7	Sagittal crest≫rostral chamber	High and domed	Moderately developed and deep	-	Minute, external

Freq. = peak (time expansion or full-spectrum detector) or maximum (ANABAT detector) frequency; NLW = nasal width; FL = forearm length; GSL = greatest skull length; CCL = condylo-canine skull length.

* Echolocation frequency of holotype not measured but assumed on basis of two released individuals of the same species at Mt Mabu recorded during the same month (October 2008).

** We assume the holotype and paratype specimens from the Taita District may have had a CF frequency of *ca* 42 kHz based on recent recordings from the type locality.

*** The holotype was not recorded but we assume a CF frequency of 35–38 kHz based on recordings of four bats taken from nearby localities in northern Mozambique.

#### Paratypes

DM 7886 (adult male, alcohol skin and skull, collected by L. Cohen on 27 September 2004 from type locality), DM11620, DM 11919, DM 11918 (adult males, alcohol skin and skull, collected by S. Stoffberg and L. Cohen on 27 September 2009).

#### Description

Dorsal and ventral colour similar to *R. hildebrandtii* s.s., but larger in noseleaf, skull and external dimensions as described above. The noseleaf shows the typical low, rounded shape of the connecting process in lateral view, in the holotype (Fig. 8b) and in other specimens examined (e.g. Fig. 8a). Lower lip with single mental groove. The robust skull is represented in the mean cranial dimensions (Table 5; Fig. 5). In lateral view the skull has a prominent rostral chamber that extends to the same height as the sagittal crest; albeit the cranium is distinctly elongated and flattened in lateral profile (Fig. 6). In dorsal view the V-shaped inter-orbital basin formed by the supraorbital ridges is very prominent, with the anterior root of the sagittal crest displaced posteriorly (Fig. 6).

#### Distribution

Known only from three closely-spaced localities in Mpumalanga Province of South Africa in the vicinity of Nelspruit.

#### Ecology

The type locality of Barberton is located in the Savanna Biome [114] at 690 m, close to the border of the Grassland Biome. Two nearby additional localities (Sudwala and Mayo Mines) are located in the Grassland Biome (Mesic Highveld Grassland) at altitudes between 900 and 1100 m.

#### Etymology

We selected the specific epithet to recognize Lientjie Cohen who collected the type specimen. She has contributed significantly to the conservation of bats in South Africa, particularly in Mpumalanga Province. The species name combines the surname Cohen and genitive singular case-ending “ae” indicative of feminine gender.

#### Specimens examined

See Table S1.

### *Rhinolophus mabuensis* new species

urn:lsid:zoobank.org:act:C205928E-D72C-40D5-9647-FDC9391CB1A6

Mount Mabu Horseshoe Bat

Fig. 4, 4, Fig. S1, Table 3, 4, Table S1, Appendix S1

#### Holotype

DM 10842; adult male, alcohol skin, skull and prepared baculum, collected by M. Curran and M. Kopp, 13 October 2008.

#### Type locality

Mt Mabu, northern Mozambique, 16° 17′ 2″S; 36° 23′ 53″E; elevation 1043 m asl. Diagnosis. Peak frequency of 37.5 and 37.9 kHz for two animals captured and released in sub-montane and montane forests at 550 m and 1000 m respectively on Mt Mabu (Curran and Kopp, personal communication). The echolocation call frequencies of the holotype from Mt Mabu and the paratype from Mt Inago were not measured. Body, cranium and noseleaf very large (FL 66.1 mm in holotype, 69.0 in paratype; GSL 29.7 mm in holotype; 30.3 mm in paratype; CCL 25.2 mm in holotype, 25.9 mm in paratype; NLW 15.0 mm in holotype, 15.3 mm in paratype; Table 5); size and position of anterior upper premolar variable; either relatively largeand situated partially within the toothrow, with gap between canine and posterior premolar (paratype) or small and located external to the toothrow (holotype). Much larger than all other members of the *R. hildebrandtii* complex, except for *R. cohenae* from which *mabuensis* can be distinguished by echolocation frequency (33 kHz in *R. cohenae*; *ca*38 kHz in *R. mabuensis*) and baculum shape (Type 2 in *mabuensis*; Type 1 in *cohenae*; Fig. 9). Genotypes of *R. mabuensis* are members of Clade 1b (Fig. 2).

#### Paratype

DM 11485 (adult female, alcohol skin and skull, collected J. Bayliss on 5 September 2009 from Mt Inago).

#### Description

External and noseleaf description and colour similar to *R. hildebrandtii* s.s., but larger in skull and external dimensions (see above). Lower lip with single mental groove. Noseleaf shape variable with profile of connecting process forming a continuous arch as typical for *R. hildebrandtii* [12] in the individual from Mt Inago, but highly distinctive in the Mt Mabu specimen, representing a small rounded bump (Fig. 8). The robust skull is reflected in the large means recorded for most cranial dimensions (Table 5). In lateral view the skull has a prominent rostral chamber which extends to the same height as the relatively weak sagittal crest. In dorsal view the V-shaped inter-orbital basin (frontal depression [12]) formed by the supraorbital ridges is very prominent and deep, and the anterior root of the sagittal crest is displaced posteriorly.

#### Distribution

Known only from two mountains in northern Mozambique but quite possibly extending to nearby Mts. Namuli, Chiperone, Mulanje and the Malawi Rift.

#### Ecology

All known specimens were associated with montane or sub-montane forest on the two mountains where they were collected.

#### Etymology

We selected the specific epithet to draw attention to the serious threats to the unique biodiversity isolated on the montane forest islands in northern Mozambique – notably Mts Mabu and Inago. None of these landforms lie within formally protected areas, and all are undergoing major habitat degradation and destruction from ever-increasing human activities - hunting, fires, timber harvesting and expanding agriculture [22]. The conservation status of this threatened biodiversity on Mts Mabu and Inago is highlighted by *R. mabuensis*, alongside recently discovered species of butterflies, crabs, snakes and chameleons [22]; [115], [116], [117], [118].

#### Specimens examined

See Table S1.

### *Rhinolophus hildebrandtii* Peters, 1878

Hildebrandt's Horseshoe Bat

Fig. 4, 4, Fig. S1, Table 3, 4, Table S1, Appendix S1

#### Holotype

ZMB 5378 (male collected by S. Hildebrandt in July 1877 from Ndi, Taita, Kenya). Cotype ZMB 5379 (male collected by S. Hildebrandt in July 1877 from Ndi, Taita, Kenya).

#### Type locality

Ndi, Taita, Kenya, 2° 46′ 43″S; 38° 46′ 18″E; elevation 390 m asl.

#### Diagnosis

Individuals from two localities in Kenya, including the type locality of Taita District, had a peak frequency of 42 kHz [15]. Intermediate in body and cranial size, with the type and co-type overlapping in cranial dimensions with members of Clade 2 from which they are however easily distinguished genetically based on sequences from three mitochondrial and one nuclear gene (cytochrome b uncorrected genetic divergence 7.7–9.0%). Although there is minor overlap in individual cranial and forearm variables (Table 5), they can be distinguished completely by PCA of cranial dimensions from other members of Clade1 which are distinctly smaller (Lineages 1d, 1e) or larger (Lineages 1a, 1b) (Fig. 5b). The anterior upper premolar, when present is moderate-sized but situated external to the toothrow (with the canine and P^4^ in contact) [12]. In this respect, they are distinguished from *cohenae* which have relatively conspicuous anterior premolars within the toothrow and *smithersi* in which the anterior premolar is small to minute and external to the toothrow. Genotypes of *R. hildebrandtii* are members of Clade 1c (Fig. 2).

#### Paratype

BM 79.1.21.1, Taita (un-sexed individual collected in 1877 by S. Hildebrandt).

#### Description

Follows the general description for the species [12]; however, *hildebrandtii* s.s. as defined here (restricted to East Africa) is intermediate-sized in most external and cranial dimensions and varies from the range of values reported previously for *R. hildebrandtii* s.l. [12], for example noseleaf width in nine East African specimens examined varied from 10–14 mm, rather than 12–15 mm given by previous description [12].

#### Distribution

As here defined, and based only on limited material examined from Kenya and Tanzania in the Natural History Museum, London and Berlin Museum in this study (n = 11; mensural data reported in Table 5), we restrict the distribution of *hildebrandtii* s.s. to East Africa, whilst recognising that northern populations in Democratic Republic of Congo, Ethiopia and southern Sudan (now South Sudan) are possibly referable to this species (as discussed by Koopman 1975, [13]. Moreover, given that two individuals with peak frequency of 37 kHz from Lutope-Ngolangola in NW Zimbabwe grouped genetically with individuals from coastal Mozambique assigned to Clade 2, it seems probable that *hildebrandtii* as here defined (coinciding with the divergent Lineage 1c) does not occur in southern Africa. Nevertheless, the taxonomic affinities of material from Zambia and Malawi remain unclear as craniometric analysis was unable to differentiate molecular clades and lineages at this scale (Fig. S2). Future studies need to incorporate molecular and bacular data from central, equatorial and northeastern Africa (particularly Zambia, Malawi, DRC and Ethiopia) to test the species limits of *hildebrandtii*. It is possible that yet more cryptic species remain to be discovered.

#### Ecology

This species was recorded from montane and submontane forests in the Usambara Mountains of the Eastern Arc Range in Tanzania; means and ranges given for external and cranial measurements for four individuals from this population conform very closely to our data [119].

### *Rhinolophus smithersi* new species

urn:lsid:zoobank.org:act:E78B0F44-7534-4991-975A-F176CB668DDE

Smithers's Horseshoe Bat

Fig. 4, Fig. S1, Table 3, 4, Table S1, Appendix S1

#### Holotype

NMZB 33647, alcohol and cleaned skull. Collector Number FWC 4764. Female collected 26 October 2000 by F. P. D. Cotterill and P. J. Taylor. Skull and mandible in good condition.

#### Type locality

Ngolangola Gorge at confluence with Lutope River, Sebungwe District, Gokwe Communal Land, NW Zimbabwe; 18° 17′05″S; 28° 05′ 00″E; elevation 1000 m asl.

#### Diagnosis

Peak echolocation frequency at 44–46 kHz which is considerably higher than all other members of this species-complex (33–42 kHz). Very small-cranium (mean CCL 23.4 mm; 23.0–24.3 mm; n = 6; 23.2 mm in holotype), the holotype having a disproportionately wide noseleaf (14 mm; 53% of skull length) (although this is not as pronounced in the series from Pafuri, NE South Africa; mean noseleaf width 10.7 mm; 39% of skull length), it is immediately distinguishable from other members of the *R. hildebrandtii* complex on size and peak frequency (Figs. 3; 4; Table 5). Genotypes of *R. smithersi* are members of Clades 1d and 1e (Fig. 2, Fig. S1).

#### Paratype

NMZB 33652, male alcohol and cleaned skull, Collector Number FWC 4769. Collected 26th October 2000 by F.P.D. Cotterill and P.J. Taylor from Lutope-Ngolangola Gorge, Zimbabwe.

#### Description

Apart from its distrinctly smaller size, conforming generally to the keys and published description for *R. hildebrandtii* s.l. [12], i.e. a relatively large-sized (forearm length 60.7 mm in holotype) bat with medium or large ears, wide horseshoe (10–14 mm) covering the muzzle, sella constricted at its proximal third, almost parallel-sided above and having long hairs, lancet relatively long and straight-sized, lower lip with single median groove, upper parts greyish-brown, with individual hairs long and unicoloured, underparts same colour or very slightly paler, skull large and heavily built. Lower lip with single mental groove. The sagittal crest is very well developed in relation to the nasal inflation and the rostral depression is weakly developed. The anterior premolar is small-sized and external to the toothrow (Table 5).

#### Distribution

Known from Lutope-Ngolangola Gorge south of the Zambezi Escarpment in NW Zimbabwe and also from Pafuri in the Limpopo Valley in the foothills of the Soutpansberg Mountains of northern Limpopo Province, South Africa but likely more widespread across savanna woodlands of the Limpopo and Zambezi valleys, and their escarpments (the Gwembe horst, and the Soutpansberg and Waterberg Mountains, respectively). Accurate delimitation of this species' range is subject to further collecting and reappraisal of existing museum material. This applies to papers by M.B. Fenton and co-workers based on intensive studies of the bat fauna, which included populations of this species complex in the Sengwa Wildlife Research Area, only ∼5 km north of the type locality of *R. smithersi* [120], [121], [122], [123], [124], [125], [126]. These all reported the largest rhinolophid studied as *R. hildebrandtii*, but these data would refer to both *R. mossambicus* and *R. smithersi*.

#### Ecology

Poorly known; this species occurs sympatrically with *R. mossambicus* at one locality in miombo savanna on Karoo Sandstone, dominated by trees of *Brachystegia glaucescens*, and including large specimens of baobabs, *Adansonia digitata*; albeit much of the landscape has been converted to cotton fields. More diverse riparian woodland fringes the Lutope and Ngolanola rivers as well as along the Limpopo River at Pafuri. Daylight roosts were not located but these bats could use caves in the sandstone cliffs and/or hollows in baobabs.

#### Etymology

We selected the specific epithet in recognition of the late Reay Henry Noble Smithers (1907–1987), former Director of the National Museums of Zimbabwe, prodigious collector and researcher of mammals including bats, and author of important regional texts on the mammalogy of Botswana, Zimbabwe and Mozambique, notably his definitive monograph – *The Mammals of the Southern African Subregion* subsequently updated and revised [127]. The species name combines the surname Smithers and genitive singular case-ending “i” indicative of masculine gender.

#### Specimens examined

See Table S1.

### *Rhinolophus mossambicus* new species

urn:lsid:zoobank.org:act:0D97DC39-F9CD-415E-A7AE-4DCF70C807B4

Mozambican Horseshoe Bat

Fig. 4, 4, Fig. S1, Table 3, 4, Table S1, Appendix S1

#### Holotype

DM8578, male, alcohol skin, skull and baculum, collected by A. Monadjem on 1 July 2006. Skull and mandible in good condition.

#### Type locality

Niassa Game Reserve (Maputo Camp), northern Mozambique, 12° 10′56″S; 37° 33′ 00″E; elevation 489 m asl.

#### Diagnosis

Peak frequency between 35–38 kHz (not recorded in holotype but inferred from members of the same genetic clade). Members of this cryptic species are genetically distinct from *R. hildebrandtii* s.s. (7.7–9.0% cytochrome b uncorrected divergence; Clade 2 in Fig. 2 and Fig. S1). Noseleaf with typical low rounded profile of connecting process in the holotype (Fig. 8d) although slight variation was observed among members of this species with one specimen having a higher and rounded shape (Fig. 8f). Skull intermediate-sized (GSL 28.5 mm; CCL 25.0 mm in the holotype; range of GSL 27–29 mm; CCL 24–26 mm; Table 5) and can be distinguished from larger (*cohenae*, *mabuensis*) and smaller (*smithersi*) species, although it is overlapped by measurements of the type (GSL 27.4 mm) and cotype (GSL 27.6 mm) of *R. hildebrandtii* (Table 5). Frontal depression reduced in holotype and five other members of this species examined; sagittal crest very pronounced, much higher than rostral chamber in lateral profile and commencing just posterior to the reduced frontal depression; in this respect it can be distinguished from *cohenae* and *mabuensis* in which the sagittal crest is reduced, and starts further back mid-dorsally in the inter-orbital region behind a well-developed frontal depression. The baculum conforms to the “Type 2” (shaft wide in dorsal view and horizontal in lateral view with rounded tip) and measures 3.14 mm in total length in the holotype (Fig. 9; Table 5). The anterior upper premolar is minute and external to the toothrow (in the holotype and three additional specimens examined; in a fourth (DM 11276) the anterior premolar was absent), as found also in *R. eloquens* but not in any other members of the *hildebrandtii* complex. There is a single mental groove in the holotype and in three other members of this species examined. Genotypes classify *R. mossambicus* in Clade 2.

#### Paratypes

DM 8577 adult female alcohol and skull from Namapa, northern Mozambique; DM 8579 adult male alcohol, skull and baculum from Chinizuia, central Mozambique; DM 8580 adult male alcohol, skull and baculum from Gorongosa Caves, central Mozambique; DM 11276, adult female alcohol and skull from Gerhard's Cave, southern Mozambique.

#### Description

Generally conforms to the previously published keys and description [12], i.e. a large-sized bat (forearm 60–65 mm; 63.9 mm in holotype) with medium or large ears (28–37 mm; 32.6 mm in holotype), wide horseshoe (13–14.2 mm; 14.2 mm in holotype) covering the muzzle, sella constricted at its proximal third, almost parallel-sided above and having long hairs, lancet relatively long and straight-sized, lower lip with single median groove, upper parts greyish-brown, with individual hairs long and unicoloured, underparts same colour or very slightly paler, skull large and heavily built (Fig. 9).

#### Distribution

Based on genetic data, the species is known from five localities in Mozambique from altitudes of <500 m (Fig. 1: Chinizuia Forest, Gerhard's Cave, Gorongosa Caves, Namapa and Niassa Game Reserve) and one locality, Lutope-Ngolangola Confluence in NW Zimbabwe. These scattered records across the subregion suggest that *R. mossambicus* is widespread across the savanna biome of Zimbabwe and Mozambique. Its overall range awaits elucidation (Fig. 2).

#### Ecology

Associated with the southern savanna biome. Based on five localities in Mozambique, its range extends from 60–500 m, and to 1000 m in NW Zimbabwe.

#### Etymology

The name denotes the country of origin (Mozambique) of the type specimen of this species. Additional sequenced specimens from Mozambique suggest that the species is widely distributed throughout this country. However *R. mossambicus* was not recorded on montane islands (Mts Mabu and Inago) to which populations of *R. mabuensis* are restricted.

#### Specimens examined

See Table S1.

## Supporting Information

Table S1 **Collecting details of specimens of the** ***Rhinolophus hildebrandtii*** **complex used in integrated morphological, molecular and acoustic analyses.** All specimens listed were included in conventional craniometric analysis; those underlined were also included in geometric morphometric analysis. For voucher specimens used in molecular analyses, clade assignments (based on cytb data in Fig. 2) are given. For specimens with acoustic data, the peak frequency (Freq.) of the CF component of the echolocation calls is given. Locality numbers correspond with those in Fig. 2. Institutional acronyms are as follows: DM: Durban Natural Science Museum, Durban, South Africa; TM: Ditsong National Natural History Museum (formerly Transvaal Museum), Pretoria, South Africa; NMZB: Natural History Museum of Zimbabwe, Bulawayo; EBD: Estación Biológica de Doñana, Sevilla, Spain; FMNH: Field Museum of Natural History, Chicago, Illinois; TTU: Museum of Texas Tech University, Lubbock, Texas, ZMB: Zoologisches Museum, Berlin. Country codes as follows: MZ = Mozambique; SA = South Africa; ZW = Zimbabwe. * - specimens from Pafuri used for morphometric analysis were obtained from historical collections in the TM; however, frequencies and clade assignment for this population were based on recent analyses without voucher specimens (Fig. 2; Table 3, Appendix S1). ** - although molecular and acoustic data were not available directly for type specimens of *R. hildebrandtii*, molecular and acoustic data are available from the type locality of Taita District in Kenya as well as additional localities in Kenya to allow the confident assignment of type specimens to Clade 1c (Fig. 2) and to a frequency of 42 kHz [15]. *** - although molecular data were not available for the type series of *R. eloquens*, DNA sequences from the Malindi District of Kenya based on confidently identified material in the Field Museum of Natural History allowed us to assign the species to Clade 3 (Fig. 2, Appendix S1). Revised taxonomic names for each clade are given; where molecular sequences were not available, morphometric criteria were used to assign taxon names (see Taxonomic Conclusions for detailed species accounts).
(DOCX)

Table S2 **Uncorrected p sequence distances for the control region data set.** Taxon names refer to those in Appendix S1. The clades referred to in the text are also given.
(DOCX)

Appendix S1 **Specimens of the** ***Rhinolophus hildebrandtii*** **complex used in molecular analyses, with GenBank Accession numbers for taxa sequenced for mtDNA markers: control region (CR), 12S, cytochrome b (Cytb) and/or the nDNA marker Chd1.**
(DOC)

Figure S1 **The consensus topology of trees (excluding burnin) sampled in a three million Bayesian analysis (MrBayes).** Bayesian posterior probabilities (PP) and parsimony bootstrap support (BT) are provided above major nodes (PP/BT). The three topologies are as follows: A – Chd1 gene, B - 12S gene, and C – control region. The three main clades and three lineages referred to in the text are indicated on each topology. Taxa abbreviations are RH – *Rhinolophus hildebrandtii*, RcfH – *R*. cf. *hildebrandtii*, and RE – *R. eloquens*, and numbers refer to the field or museum accession numbers and correspond to those in Appendix S1. Numbers in parentheses correspond to place names in Table S1 and Fig. 2. *Rhinolophus clivosus* was used as the outgroup.
(TIF)

Figure S2 **Canonical variates analysis of six craniometric variables in ten Operational Taxonomic Units (OTUs) based on a sample of 171 adult male skulls of** ***R. hildebrandtii*** **and** ***R. eloquens*** **.** Clades and lineages defined on molecular grounds indicated by 1a (Clade 1: Mpumalanga Province, SA), 1c (Clade 1: Pafuri, Kruger NP, SA) and 2a (Clade 2). Codes indicate species and OTUs as follows: q = *R. eloquens* (including type series); balance of OTUs represent *R. hildebrandtii*: s = southern Zimbabwe; c = central Zimbabwe; e = eastern Zimbabwe; n = northern Zimbabwe; za = Zambia+Malawi; ea = Kenya and Tanzania (including type and co-type from Kenya). 95% confidence ellipses shown for each group.
(TIF)

Figure S3 **Plots of peak frequency of the CF component of echolocation calls versus condylo-canine skull length (CCL) (a) and altitude (b); and (c) means and 95% confidence limits of relative importance after 1000 bootstrap replications (using “lmg” method in R) for ten predictor variables used in complex linear model (Model 5 in text) to explain variation in CF frequency in** ***R. hildebrandtii*** **.** Alt = altitude group (<600 m or >600 m); CCL = condylocanine skull length; FL = forearm length; Clade = membership of molecular clades and subcaldes (Fig. 3); NH = height of rostral chamber; NW = width of rostral chamber; NL = length of skull from occiput to anterior of rostral chamber; RH = relative humidity.
(TIF)

Figure S4 **Drawings of bacula from: (a) Marondera, Zimbabwe (NMZB 62133–62139; 62141–62142; 62144–621477; except where noted otherwise, top = dorsal; bottom = lateral), Esigodeni, Zimbabwe (b; NMZB 80237; total length = 4.6 mm) and Luangwa, Zambia (c; NMZB 62149; total length = 2.6 mm).** In (b) top = dorsal, middle = lateral and bottom = ventral and (c) top = dorsal, middle = ventral and bottom = lateral.
(TIF)
