# Supplementary material for: Four New Bat Species (Rhinolophus hildebrandtii Complex) Reflect Plio-Pleistocene Divergence of Dwarfs and Giants across an Afromontane Archipelago
Source: PLoS One. 2012 Sep 12;7(9):e41744. doi: 10.1371/journal.pone.0041744 (PMC3440430; doi:10.1371/journal.pone.0041744)
Supplement: Table S1 — Collecting details of specimens of the Rhinolophus hildebrandtii complex used in integrated morphological, molecular and acoustic analyses. All specimens listed were included in conventional craniometric analysis; those underlined were also included in geometric morphometric analysis. For voucher specimens used in molecular analyses, clade assignments (based on cytb data in Fig. 2) are given. For specimens with acoustic data, the peak frequency (Freq.) of the CF component of the echolocation calls is given. Locality numbers correspond with those in Fig. 2. Institutional acronyms are as follows: DM: Durban Natural Science Museum, Durban, South Africa; TM: Ditsong National Natural History Museum (formerly Transvaal Museum), Pretoria, South Africa; NMZB: Natural History Museum of Zimbabwe, Bulawayo; EBD: Estación Biológica de Doñana, Sevilla, Spain; FMNH: Field Museum of Natural History, Chicago, Illinois; TTU: Museum of Texas Tech University, Lubbock, Texas, ZMB: Zoologisches Museum, Berlin. Country codes as follows: MZ = Mozambique; SA = South Africa; ZW = Zimbabwe. * - specimens from Pafuri used for morphometric analysis were obtained from historical collections in the TM; however, frequencies and clade assignment for this population were based on recent analyses without voucher specimens (Fig. 2; Table 3, Appendix S1). ** - although molecular and acoustic data were not available directly for type specimens of R. hildebrandtii, molecular and acoustic data are available from the type locality of Taita District in Kenya as well as additional localities in Kenya to allow the confident assignment of type specimens to Clade 1c (Fig. 2) and to a frequency of 42 kHz [15]. *** - although molecular data were not available for the type series of R. eloquens, DNA sequences from the Malindi District of Kenya based on confidently identified material in the Field Museum of Natural History allowed us to assign the species to Clade 3 (Fig. 2, Appendix S1). Revised taxonomic names f [file pone.0041744.s001.docx]

**Table S1.** Collecting details of specimens of the *Rhinolophus hildebrandtii* complex used in integrated morphological, molecular and acoustic analyses.

| Species and Museum number | Locality (numbers in parentheses refer to those shown in Fig. 1) | Clade (from Fig. 2) | Revised taxon name | Latitude | Longitude | Sex | Alt. (m) | Freq. (kHz) |
| --- | --- | --- | --- | --- | --- | --- | --- | --- |
| *Rhinolophus hildebrandtii* s.l. | |  |  |  |  |  |  |  |
| DM 8626 | SA: Mpumalanga, Barberton, Mountainland Nature Reserve, 68km SE Sudwala (1) | - | *cohenae* sp. nov. | -25.43 | 31.16 | M | 690 | 33.0 |
| DM 7886 | “ “ | 1a | *cohenae* sp. nov. | -25.43 | 31.16 | M | 690 | 33.0 |
| DM 11620 | “ “ | 1a | *cohenae* sp. nov. | -25.43 | 31.16 | M | 690 | 33.9 |
| DM 11619 | “ “ | 1a | *cohenae* sp. nov. | -25.43 | 31.16 | M | 690 | 33.0 |
| DM 11618 | “ “ | 1a | *cohenae* sp. nov. | -25.43 | 31.16 | M | 690 | 33.4 |
| TM 46641 | SA: Sudwala Mines (2) | - | *cohenae* sp. nov. | -25.39 | 30.68 | F | 902 | - |
| DM 11558 | SA: Sudwala Mines, 40 km W Nelspruit (2) | 1a | *cohenae* sp. nov. | -25.39 | 30.68 | M | 902 | 32.8 |
| DM 11557 | “ “ | 1a | *cohenae* sp. nov. | -25.39 | 30.68 | M | 902 | 32.6 |
| DM 11559 | SA: Mayo, 20km W Nelspruit (3) | 1a | *cohenae* sp. nov. | -25.37 | 30.74 | M | 1015 | 32.4 |
| DM 11560 | “ “ | 1a | *cohenae* sp. nov. | -25.37 | 30.74 | M | 1015 | 33.3 |
| DM 11485 | MZ: Mt Inago (4) | 1b | *mabuensis* sp. nov. | -15.13 | 37.65 | F | 1000 | ? |
| DM 10842 | MZ: Mt Mabu (5) | 1b | *mabuensis* sp. nov. | -16.28 | 36.40 | F | 1043 | 37.7 |
| TM 40836 | SA: Pafuri, Kruger NP (6) | *1d | *smithersi* sp. nov. | -22.44 | 31.18 | M | 299 | *44.0 |
| TM 42007 | “ “ | *1d | *smithersi* sp. nov. | -22.44 | 31.18 | M | 299 | *44.0 |
| TM 46435 | “ “ | *1d | *smithersi* sp. nov. | -22.44 | 31.18 | M | 299 | *44.0 |
| TM 40831 | “ “ | *1d | *smithersi* sp. nov. | -22.44 | 31.18 | M | 299 | *44.0 |
| TM 41997 | “ “ | *1d | *smithersi* sp. nov. | -22.44 | 31.18 | M | 299 | *44.0 |
| DM 8577 | MZ: Namapa (7) | 2 | *mossambicus* sp. nov. | -13.49 | 39.78 | F | 244 | 38.5 |
| DM 8578 | MZ: Niassa GR, Maputo Camp (8) | 2 | *mossambicus* sp. nov. | -12.18 | 37.55 | M | 489 | ? |
| DM 8579 | MZ: Chinizuia Forest (9) | 2 | *mossambicus* sp. nov. | -18.98 | 35.05 | F | 154 | 37.6 |
| DM 8580 | MZ: Gorongoza Caves (10) | 2 | *mossambicus* sp. nov. | -18.56 | 34.87 | M | 238 | 34.9 |
| DM 11276 | MZ: Gerhard's cave (11) | 2 | *mossambicus* sp. nov. | -21.67 | 34.86 | F | 68 | 38 |
| NMZB 33644 | ZW: Lutope Ngolangola Confluence, Sebungwe (12) | 2 | *mossambicus* sp. nov. | -18.28 | 28.08 | M | 1002 | 37.0 |
| NMZB 33646 | “ “ | - | *mossambicus* sp. nov. | -18.28 | 28.08 | M | 1002 | 37.3 |
| NMZB 33647 | “ “ | - | *smithersi*  sp. nov. | -18.28 | 28.08 | F | 1002 | 46.0 |
| NMZB 33648 | “ “ | 2 | *mossambicus* sp. nov. | -18.28 | 28.08 | M | 1002 | 37.0 |
| NMZB33652 | “ “ | 1e | *smithersi* sp. nov. | -18.28 | 28.08 | M | 1002 | ? |
| NMZB 33649 | “ “ | - | *mossambicus* sp. nov. | -18.28 | 28.08 | M | 1002 | ? |
| NMZB 33650 | “ “ | - | *mossambicus* sp. nov. | -18.28 | 28.08 | F | 1002 | ? |
| NMZB33651 | “ “ | - | *mossambicus* sp. nov. | -18.28 | 28.08 | M | 1002 | 37.0 |
| NMZB 33653 | “ “ | - | *mossambicus* sp. nov. | -18.28 | 28.08 | F | 1002 | ? |
| NMZB 33654 | “ “ | - | *mossambicus* sp. nov. | -18.28 | 28.08 | M | 1002 | ? |
| NMZB 33669 | “ “ | - | *mossambicus* sp. nov. | -18.28 | 28.08 | M | 1002 | 37.0 |
| NMZB 33670 | “ “ | - | *mossambicus* sp. nov. | -18.28 | 28.08 | M | 1002 | 37.0 |
| NMZB 33671 | “ “ | - | *mossambicus* sp. nov. | -18.28 | 28.08 | M | 1002 | 37.9 |
| NMZB 33672 | “ “ | - | *mossambicus* sp. nov. | -18.28 | 28.08 | M | 1002 | 37.0 |
| NMZB 33673 | “ “ | - | *mossambicus* sp. nov. | -18.28 | 28.08 | M | 1002 | 37.1 |
| NMZB 33674 | “ “ | - | *mossambicus* sp. nov. | -18.28 | 28.08 | M | 1002 | 36.5 |
| NMZB 33675 | “ “ | - | *mossambicus* sp. nov. | -18.28 | 28.08 | M | 1002 | 37.0 |
| NMZB 33676 | “ “ | - | *mossambicus* sp. nov. | -18.28 | 28.08 | M | 1002 | 37.0 |
| NMZB 33679 | “ “ | - | *mossambicus* sp. nov. | -18.28 | 28.08 | M | 1002 | ? |
| NMZB 33680 | “ “ | - | *mossambicus* sp. nov. | -18.28 | 28.08 | M | 1002 | ? |
| NMZB 33682 | “ “ | - | *mossambicus* sp. nov. | -18.28 | 28.08 | M | 1002 | ? |
| NMZB 33683 | “ “ | - | *mossambicus* sp. nov. | -18.28 | 28.08 | M | 1002 | ? |
| NMZB 33706 | “ “ | - | *mossambicus* sp. nov. | -18.28 | 28.08 | F | 1002 | 37.0 |
| NMZB 33707 | “ “ | - | *mossambicus* sp. nov. | -18.28 | 28.08 | M | 1002 | 37.0 |
| NMZB 33710 | “ “ | - | *mossambicus* sp. nov. | -18.28 | 28.08 | M | 1002 | 37.0 |
| ZMB5378  (Holotype) | Kenya: Ndi, Taita (13) | **1c | *hildebrandtii* s.s. | -2.78 | 38.77 | M | 390 | **42 |
| ZMB5379  (Paratype) | Kenya: Ndi, Taita | **1c | *hildebrandtii* s.s. | -2.78 | 38.77 | M | 390 | **42 |
| *R. eloquens* | | - |  |  |  |  |  |  |
| BM99.8.4.5  (Paratype) | Uganda: Entebbe (14) | ***3 | *eloquens* | 0.044 | 32.46 | ? | 1191 | ? |
| BM64.2078  (Topotype) | Uganda: Entebbe | ***3 | *eloquens* | 0.044 | 32.46 | ? | 1191 | ? |
| BM99.8.4.3 | Uganda: Entebbe | ***3 | *eloquens* | 0.044 | 32.46 | M | 1191 | ? |
| BM99.8.4.4  (Holotype) | Uganda: Entebbe | ***3 | *eloquens* | 0.044 | 32.46 | ? | 1191 | ? |
| BM99.8.4.6 | Uganda: Entebbe | ***3 | *eloquens* | 0.044 | 32.46 | ? | 1191 | ? |

All specimens listed were included in conventional craniometric analysis; those underlined were also included in geometric morphometric analysis. For voucher specimens used in molecular analyses, clade assignments (based on cytb data in Fig. 2) are given. For specimens with acoustic data, the peak frequency (Freq.) of the CF component of the echolocation calls is given. Locality numbers correspond with those in Fig. 1. Institutional acronyms are as follows: DM: Durban Natural Science Museum, Durban, South Africa; TM: Ditsong National Natural History Museum (formerly Transvaal Museum), Pretoria, South Africa; NMZB: Natural History Museum of Zimbabwe, Bulawayo; EBD: Estación Biológica de Doñana, Sevilla, Spain; FMNH: Field Museum of Natural History, Chicago, Illinois; TTU: Museum of Texas Tech University, Lubbock, Texas, ZMB: Zoologisches Museum, Berlin. Country codes as follows: MZ = Mozambique; SA = South Africa; ZW = Zimbabwe. * - specimens from Pafuri used for morphometric analysis were obtained from historical collections in the TM; however, frequencies and Clade assignment for this population were based on recent analyses without voucher specimens (Fig. 2; Table 2, Appendix 1). ** - although molecular and acoustic data were not available directly for type specimens of *R. hildebrandtii*, molecular and acoustic data are available from the type locality of Taita District in Kenya as well as additional localities in Kenya to allow the confident assignment of type specimens to Clade 1c (Fig. 2) and to a frequency of 42 kHz [15]. *** - although molecular data were not available for the type series of *R. eloquens*, DNA sequences from the Malindi District of Kenya based on confidently identified material in the Field Museum of Natural History allowed us to assign the species to Clade 3 (Fig. 2, Appendix 1). Revised taxonomic names for each clade are given; where molecular sequences were not available, morphometric criteria were used to assign taxon names (see Taxonomic Conclusions for detailed species accounts).
