# Supplementary material for: Four New Bat Species (Rhinolophus hildebrandtii Complex) Reflect Plio-Pleistocene Divergence of Dwarfs and Giants across an Afromontane Archipelago
Source: PLoS One. 2012 Sep 12;7(9):e41744. doi: 10.1371/journal.pone.0041744 (PMC3440430; doi:10.1371/journal.pone.0041744)
Supplement: Table S2 — Uncorrected p sequence distances for the control region data set. Taxon names refer to those in Appendix S1. The clades referred to in the text are also given. (DOCX) [file pone.0041744.s002.docx]

**Table S2**. Uncorrected p sequence distances for the control region data set. Taxon names refer to those in Appendix 1. The clades referred to in the text are also given.

|  |  | **RH SS133** | **RH SS61** | **RH SS62** | **RH SS63** | **RcfH DM11276** | **RcfH DM8577** | **RcfH DM8578** | **RcfH DM8580** | **RcfH DM8579** | **RCL DM11484** | **RCL DM11483** |
| --- | --- | --- | --- | --- | --- | --- | --- | --- | --- | --- | --- | --- |
| **RH SS133** | Clade 1a |  |  |  |  |  |  |  |  |  |  |  |
| **RH SS61** | Clade 1a | 0.000 |  |  |  |  |  |  |  |  |  |  |
| **RH SS62** | Clade 1a | 0.000 | 0.000 |  |  |  |  |  |  |  |  |  |
| **RH SS63** | Clade 1a | 0.000 | 0.000 | 0.000 |  |  |  |  |  |  |  |  |
| **RcfH DM11276** | Clade 2 | 0.103 | 0.101 | 0.103 | 0.101 |  |  |  |  |  |  |  |
| **RcfH DM8577** | Clade 2 | 0.094 | 0.092 | 0.094 | 0.092 | 0.017 |  |  |  |  |  |  |
| **RcfH DM8578** | Clade 2 | 0.094 | 0.092 | 0.094 | 0.092 | 0.023 | 0.013 |  |  |  |  |  |
| **RcfH DM8580** | Clade 2 | 0.099 | 0.097 | 0.099 | 0.097 | 0.008 | 0.013 | 0.019 |  |  |  |  |
| **RcfH DM8579** | Clade 2 | 0.099 | 0.097 | 0.099 | 0.097 | 0.008 | 0.013 | 0.019 | 0.000 |  |  |  |
| **RCL DM11484** | Outgroup | 0.111 | 0.111 | 0.111 | 0.111 | 0.120 | 0.115 | 0.118 | 0.116 | 0.116 |  |  |
| **RCL DM11483** | Outgroup | 0.116 | 0.117 | 0.116 | 0.117 | 0.128 | 0.122 | 0.126 | 0.128 | 0.128 | 0.019 |  |
| **RH DM11485** | Clade 1b | 0.027 | 0.027 | 0.027 | 0.027 | 0.099 | 0.090 | 0.090 | 0.095 | 0.095 | 0.101 | 0.115 |
| **RH DM11559** | Clade 1a | 0.000 | 0.000 | 0.000 | 0.000 | 0.103 | 0.094 | 0.094 | 0.099 | 0.099 | 0.111 | 0.116 |
| **RH DM11557** | Clade 1a | 0.000 | 0.000 | 0.000 | 0.000 | 0.103 | 0.094 | 0.094 | 0.099 | 0.099 | 0.111 | 0.116 |
| **RH DM11560** | Clade 1a | 0.000 | 0.000 | 0.000 | 0.000 | 0.103 | 0.094 | 0.094 | 0.099 | 0.099 | 0.111 | 0.116 |
| **RH DM11558** | Clade 1a | 0.000 | 0.000 | 0.000 | 0.000 | 0.103 | 0.094 | 0.094 | 0.099 | 0.099 | 0.111 | 0.116 |
| **RH DM11620** | Clade 1a | 0.000 | 0.000 | 0.000 | 0.000 | 0.103 | 0.094 | 0.094 | 0.099 | 0.099 | 0.111 | 0.116 |
| **RH DM11618** | Clade 1a | 0.000 | 0.000 | 0.000 | 0.000 | 0.103 | 0.094 | 0.094 | 0.099 | 0.099 | 0.111 | 0.116 |
| **RH DM11619** | Clade 1a | 0.000 | 0.000 | 0.000 | 0.000 | 0.103 | 0.094 | 0.094 | 0.099 | 0.099 | 0.111 | 0.116 |
| **RH SS132** | Clade 1a | 0.000 | 0.000 | 0.000 | 0.000 | 0.101 | 0.092 | 0.092 | 0.098 | 0.098 | 0.111 | 0.117 |
| **RH DM10842** | Clade 1b | 0.029 | 0.029 | 0.029 | 0.029 | 0.094 | 0.084 | 0.084 | 0.090 | 0.090 | 0.107 | 0.116 |
| **RH DM7886** | Clade 1a | 0.000 | 0.000 | 0.000 | 0.000 | 0.101 | 0.092 | 0.092 | 0.098 | 0.098 | 0.109 | 0.115 |
| **RH BDP4694** | Clade 1c | 0.040 | 0.040 | 0.040 | 0.040 | 0.101 | 0.090 | 0.094 | 0.097 | 0.097 | 0.105 | 0.113 |
| **RE BDP4370** | Clade 3 | 0.086 | 0.086 | 0.086 | 0.086 | 0.096 | 0.086 | 0.086 | 0.092 | 0.092 | 0.092 | 0.105 |
| **RE BDP4329** | Clade 3 | 0.086 | 0.086 | 0.086 | 0.086 | 0.096 | 0.086 | 0.086 | 0.092 | 0.092 | 0.092 | 0.105 |
| **RE BDP4327** | Clade 3 | 0.086 | 0.086 | 0.086 | 0.086 | 0.096 | 0.086 | 0.086 | 0.092 | 0.092 | 0.092 | 0.105 |
| **RE BDP4359** | Clade 3 | 0.093 | 0.094 | 0.093 | 0.094 | 0.084 | 0.082 | 0.082 | 0.080 | 0.080 | 0.084 | 0.094 |
| **RE BDP4360** | Clade 3 | 0.082 | 0.082 | 0.082 | 0.082 | 0.096 | 0.086 | 0.086 | 0.092 | 0.092 | 0.086 | 0.099 |
| **RH BDP4646** | Clade 1c | 0.032 | 0.032 | 0.032 | 0.032 | 0.099 | 0.088 | 0.092 | 0.095 | 0.095 | 0.113 | 0.120 |
| **RH BDP4652** | Clade 1c | 0.032 | 0.032 | 0.032 | 0.032 | 0.099 | 0.088 | 0.092 | 0.095 | 0.095 | 0.113 | 0.120 |
| **RCL SS270** | Outgroup | 0.099 | 0.100 | 0.099 | 0.100 | 0.120 | 0.115 | 0.118 | 0.120 | 0.120 | 0.071 | 0.063 |
| **RcfH NMZB33648** | Clade 2 | 0.099 | 0.097 | 0.099 | 0.097 | 0.008 | 0.013 | 0.023 | 0.008 | 0.008 | 0.116 | 0.124 |
| **RH WTS2562** | Clade 1c | 0.034 | 0.034 | 0.034 | 0.034 | 0.101 | 0.088 | 0.088 | 0.097 | 0.097 | 0.107 | 0.118 |
|  |  |  |  |  |  |  |  |  |  |  |  |  |
|  |  |  |  |  |  |  |  |  |  |  |  |  |
|  |  | **RH DM11485** | **RH DM11559** | **RH DM11557** | **RH DM11560** | **RH DM11558** | **RH DM11620** | **RH DM11618** | **RH DM11619** | **RH SS132** | **RH DM10842** | **RH DM7886** |
| **RH DM11559** | Clade 1a | 0.027 |  |  |  |  |  |  |  |  |  |  |
| **RH DM11557** | Clade 1a | 0.027 | 0.000 |  |  |  |  |  |  |  |  |  |
| **RH DM11560** | Clade 1a | 0.027 | 0.000 | 0.000 |  |  |  |  |  |  |  |  |
| **RH DM11558** | Clade 1a | 0.027 | 0.000 | 0.000 | 0.000 |  |  |  |  |  |  |  |
| **RH DM11620** | Clade 1a | 0.027 | 0.000 | 0.000 | 0.000 | 0.000 |  |  |  |  |  |  |
| **RH DM11618** | Clade 1a | 0.027 | 0.000 | 0.000 | 0.000 | 0.000 | 0.000 |  |  |  |  |  |
| **RH DM11619** | Clade 1a | 0.027 | 0.000 | 0.000 | 0.000 | 0.000 | 0.000 | 0.000 |  |  |  |  |
| **RH SS132** | Clade 1a | 0.027 | 0.000 | 0.000 | 0.000 | 0.000 | 0.000 | 0.000 | 0.000 |  |  |  |
| **RH DM10842** | Clade 1b | 0.013 | 0.029 | 0.029 | 0.029 | 0.029 | 0.029 | 0.029 | 0.029 | 0.029 |  |  |
| **RH DM7886** | Clade 1a | 0.027 | 0.000 | 0.000 | 0.000 | 0.000 | 0.000 | 0.000 | 0.000 | 0.000 | 0.029 |  |
| **RH BDP4694** | Clade 1c | 0.034 | 0.040 | 0.040 | 0.040 | 0.040 | 0.040 | 0.040 | 0.040 | 0.040 | 0.036 | 0.040 |
| **RE BDP4370** | Clade 3 | 0.076 | 0.086 | 0.086 | 0.086 | 0.086 | 0.086 | 0.086 | 0.086 | 0.086 | 0.086 | 0.086 |
| **RE BDP4329** | Clade 3 | 0.076 | 0.086 | 0.086 | 0.086 | 0.086 | 0.086 | 0.086 | 0.086 | 0.086 | 0.086 | 0.086 |
| **RE BDP4327** | Clade 3 | 0.076 | 0.086 | 0.086 | 0.086 | 0.086 | 0.086 | 0.086 | 0.086 | 0.086 | 0.086 | 0.086 |
| **RE BDP4359** | Clade 3 | 0.084 | 0.093 | 0.093 | 0.093 | 0.093 | 0.093 | 0.093 | 0.093 | 0.094 | 0.082 | 0.094 |
| **RE BDP4360** | Clade 3 | 0.073 | 0.082 | 0.082 | 0.082 | 0.082 | 0.082 | 0.082 | 0.082 | 0.082 | 0.082 | 0.082 |
| **RH BDP4646** | Clade 1c | 0.034 | 0.032 | 0.032 | 0.032 | 0.032 | 0.032 | 0.032 | 0.032 | 0.033 | 0.032 | 0.033 |
| **RH BDP4652** | Clade 1c | 0.034 | 0.032 | 0.032 | 0.032 | 0.032 | 0.032 | 0.032 | 0.032 | 0.033 | 0.032 | 0.033 |
| **RCL SS270** | Outgroup | 0.095 | 0.099 | 0.099 | 0.099 | 0.099 | 0.099 | 0.099 | 0.099 | 0.100 | 0.097 | 0.098 |
| **RcfH NMZB33648** | Clade 2 | 0.095 | 0.099 | 0.099 | 0.099 | 0.099 | 0.099 | 0.099 | 0.099 | 0.098 | 0.090 | 0.098 |
| **RH WTS2562** | Clade 1c | 0.029 | 0.034 | 0.034 | 0.034 | 0.034 | 0.034 | 0.034 | 0.034 | 0.035 | 0.034 | 0.035 |
|  |  |  |  |  |  |  |  |  |  |  |  |  |
|  |  | **RH BDP4694** | **RE BDP4370** | **RE BDP4329** | **RE BDP4327** | **RE BDP4359** | **RE BDP4360** | **RH BDP4646** | **RH BDP4652** | **RCL SS270** | **RcfH MNZB33648** | **RH WTS2562** |
| **RE BDP4370** | Clade 3 | 0.082 |  |  |  |  |  |  |  |  |  |  |
| **RE BDP4329** | Clade 4 | 0.084 | 0.010 |  |  |  |  |  |  |  |  |  |
| **RE BDP4327** | Clade 5 | 0.084 | 0.010 | 0.000 |  |  |  |  |  |  |  |  |
| **RE BDP4359** | Clade 6 | 0.086 | 0.034 | 0.032 | 0.032 |  |  |  |  |  |  |  |
| **RE BDP4360** | Clade 7 | 0.078 | 0.010 | 0.011 | 0.011 | 0.029 |  |  |  |  |  |  |
| **RH BDP4646** | Clade 1c | 0.008 | 0.082 | 0.084 | 0.084 | 0.086 | 0.078 |  |  |  |  |  |
| **RH BDP4652** | Clade 1c | 0.008 | 0.082 | 0.084 | 0.084 | 0.086 | 0.078 | 0.000 |  |  |  |  |
| **RCL SS270** | Outgroup | 0.097 | 0.101 | 0.097 | 0.097 | 0.093 | 0.095 | 0.101 | 0.101 |  |  |  |
| **RcfH NMZB33648** | Clade 2 | 0.097 | 0.092 | 0.092 | 0.092 | 0.084 | 0.092 | 0.095 | 0.095 | 0.116 |  |  |
| **RH WTS2562** | Clade 1c | 0.023 | 0.076 | 0.076 | 0.076 | 0.084 | 0.073 | 0.015 | 0.015 | 0.105 | 0.097 |  |
|  |  |  |  |  |  |  |  |  |  |  |  |  |
