# Supplementary material for: Four New Bat Species (Rhinolophus hildebrandtii Complex) Reflect Plio-Pleistocene Divergence of Dwarfs and Giants across an Afromontane Archipelago
Source: PLoS One. 2012 Sep 12;7(9):e41744. doi: 10.1371/journal.pone.0041744 (PMC3440430; doi:10.1371/journal.pone.0041744)
Supplement: Appendix S1 — Specimens of the Rhinolophus hildebrandtii complex used in molecular analyses, with GenBank Accession numbers for taxa sequenced for mtDNA markers: control region (CR), 12S, cytochrome b (Cytb) and/or the nDNA marker Chd1. (DOC) [file pone.0041744.s003.doc]

**Appendix 1**. Specimens of the *Rhinolophus hildebrandtii* complex used in molecular analyses, with GenBank Accession numbers for taxa sequenced for mtDNA markers: control region (CR), 12S, cytochrome b (Cytb) and/or the nDNA marker Chd1.

| **Revised taxon name** | **Locality** | **Field code / accession number** | **Accession number** | | | | |
| --- | --- | --- | --- | --- | --- | --- | --- |
|  |  |  | **CR** | **12S** | **Chd1** | **Cyt b** | **Clade** |
| *R. clivosus* | Mt Mabu, Mozambique | DM11484 | JQ929258 | JQ929225 |  |  | - |
| *R. clivosus* | Mt Mabu, Mozambique | DM11483 | JQ929259 | JQ929224 |  |  | - |
| *R. clivosus* | Greyton, South Africa | SS212 | JN618250 |  | JQ929248 |  | - |
| *R. clivosus* | Postmasburg, South Africa | SS218 | JN618253 |  | JQ929247 |  | - |
| *R. clivosus* | Pietermaritzburg, South Africa | SS270 | JN618291 |  |  |  | - |
| *R. eloquens* | Malindi District, Kenya | BDP4370; 360241 | JQ929272 | JQ929218 | JQ929238 | JQ929281 | 3 |
| *R. eloquens* | Malindi District, Kenya | BDP4329; 360202 | JQ929273 | JQ929219 | JQ929239 | JQ929282 | 3 |
| *R. eloquens* | Malindi District, Kenya | BDP4327; 360199 | JQ929274 | JQ929220 | JQ929240 | JQ929283 | 3 |
| *R. eloquens* | Malindi District, Kenya | BDP4359; 360232 | JQ929275 | JQ929221 | JQ929241 | JQ929284 | 3 |
| *R. eloquens* | Malindi District, Kenya | BDP4360; 360233 | JQ929276 | JQ929222 | JQ929242 | JQ929285 | 3 |
| *R. mabuensis* sp. nov. | Mt Mabu, Mozambique | DM10842 | JQ929269 | JQ929205 | JQ292229 |  | 1b |
| *R. mabuensis* sp. nov. | Mt Inago, Mozambique | DM11485 | JQ929260 | JQ929214 |  | JQ929292 | 1b |
| *R. cohenae* sp. nov. | Mayo, Sudwala, South Africa | DM11559 | JQ929261 |  |  |  | 1a |
| *R. cohenae* sp. nov. | Sudwala, South Africa | DM11557 | JQ929262 |  |  |  | 1a |
| *R. cohenae* sp. nov. | Sudwala, South Africa | DM11560 | JQ929263 |  |  |  | 1a |
| *R. cohenae* sp. nov. | Mayo, Sudwala, South Africa | DM11558 | JQ929264 |  |  |  | 1a |
| *R. cohenae* sp. nov. | Barberton, South Africa | DM11620 | JQ929265 | JQ929215 | JQ929235 | JQ929293 | 1a |
| *R. cohenae* sp. nov. | Barberton, South Africa | DM11618 | JQ929266 |  |  |  | 1a |
| *R. cohenae* sp. nov. | Barberton, South Africa | DM11619 | JQ929267 |  |  |  | 1a |
| *R. cohenae* sp. nov. | Sudwala, South Africa | SS132 | JQ929268 | JQ929216 | JQ929236 | JQ929294 | 1a |
| *R. cohenae* sp. nov. | Sudwala, South Africa | SS133 | JQ929249 | JQ929217 | JQ929237 | JQ929295 | 1a |
| *R. cohenae* sp. nov. | Sudwala, South Africa | SS61 | JQ929250 | JQ929207 | JQ929231 |  | 1a |
| *R. cohenae* sp. nov. | Sudwala, South Africa | SS62 | JQ929251 |  |  |  | 1a |
| *R. cohenae* sp. nov. | Sudwala, South Africa | SS63 | JQ929252 | JQ929208 | JQ929232 |  | 1a |
| *R.cohenae* sp. nov. | Sudwala, South Africa | DM7886 | JQ929270 | JQ929206 | JQ929230 | JQ929287 | 1a |
| *R. hildebrandtii* s.s. | Morogoro Region, Tanzania | WTS2562 | JQ929280 | JQ929204 | JQ929246 | JQ929286 | 1c |
| *R. hildebrandtii* ss | Taita-Taveta District, Kenya | BDP4646; 360518 | JQ929277 | JQ929223 | JQ929243 | JQ929296 | 1c |
| *R. hildebrandtii* ss | Taita-Taveta District, Kenya | BDP4652; 360524 | JQ929278 | JQ929211 | JQ929244 | JQ929297 | 1c |
| *R. hildebrandtii* ss | Makuendi District, Kenya | BDP4694; 360562 | JQ929271 | JQ929202 | JQ929245 | JQ929298 | 1c |
| *R. smithersi* sp. nov. | Lutope, Zimbabwe | NMZB33652 |  | JQ929228 |  |  | 1e |
| *R. smithersi* sp. nov. | Pafuri, South Africa | CMR15 |  |  |  | JQ929300 | 1d |
| *R.* *mossambicus* sp. nov. | Lutope, Zimbabwe | NMZB33648 | JQ929279 | JQ929226 |  | JQ929299 | 2 |
| *R. mossambicus* sp. nov. | Lutope, Zimbabwe | NMZB33644 |  | JQ929227 |  |  | 2 |
| *R.* *mossambicus* sp. nov. | Gerhard's Cave, Mozambique | DM11276 | JQ929253 | JQ929209 | JQ929233 | JQ929301 | 2 |
| *R.* *mossambicus* sp. nov. | Namapa, Mozambique | DM8577 | JQ929254 | JQ929210 |  | JQ929288 | 2 |
| *R.* *mossambicus* sp. nov. | Niassa, Mozambique | DM8578 | JQ929255 | JQ929203 | JQ929234 | JQ929289 | 2 |
| *R.* *mossambicus* sp. nov. | Gorongoza, Mozambique | DM8580 | JQ929256 | JQ929212 |  | JQ929290 | 2 |
| *R.* *mossambicus* sp. nov. | Chinizuia, Mozambique | DM8579 | JQ929257 | JQ929213 |  | JQ929291 | 2 |

Acronyms for field / museum codes are as given in Fig. 2; SS/CMR: sequencing code for sample without voucher accessioned (e. g. wing punch); BDP: samples provided by Bruce Patterson; WTS: sample provided by Bill Stanley. Revised taxon names provided (see Taxonomic Conclusions).
