# Supplementary figures and images for: Four New Bat Species (Rhinolophus hildebrandtii Complex) Reflect Plio-Pleistocene Divergence of Dwarfs and Giants across an Afromontane Archipelago
Source: PLoS One. 2012 Sep 12;7(9):e41744. doi: 10.1371/journal.pone.0041744 (PMC3440430; doi:10.1371/journal.pone.0041744)

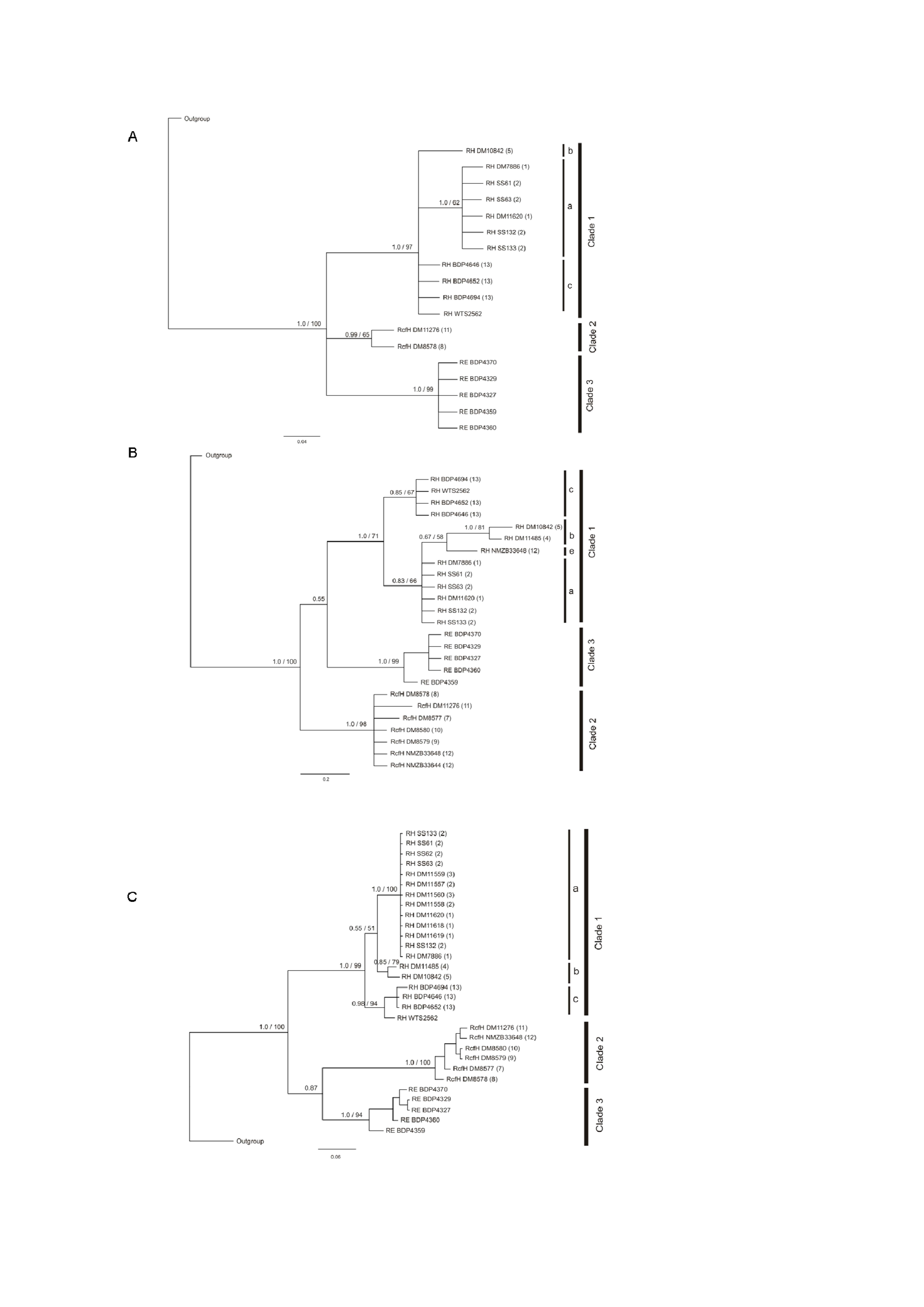

Supplement: Figure S1 — The consensus topology of trees (excluding burnin) sampled in a three million Bayesian analysis (MrBayes). Bayesian posterior probabilities (PP) and parsimony bootstrap support (BT) are provided above major nodes (PP/BT). The three topologies are as follows: A – Chd1 gene, B - 12S gene, and C – control region. The three main clades and three lineages referred to in the text are indicated on each topology. Taxa abbreviations are RH – Rhinolophus hildebrandtii, RcfH – R. cf. hildebrandtii, and RE – R. eloquens, and numbers refer to the field or museum accession numbers and correspond to those in Appendix S1. Numbers in parentheses correspond to place names in Table S1 and Fig. 2. Rhinolophus clivosus was used as the outgroup. (TIF) [file pone.0041744.s004.tif]

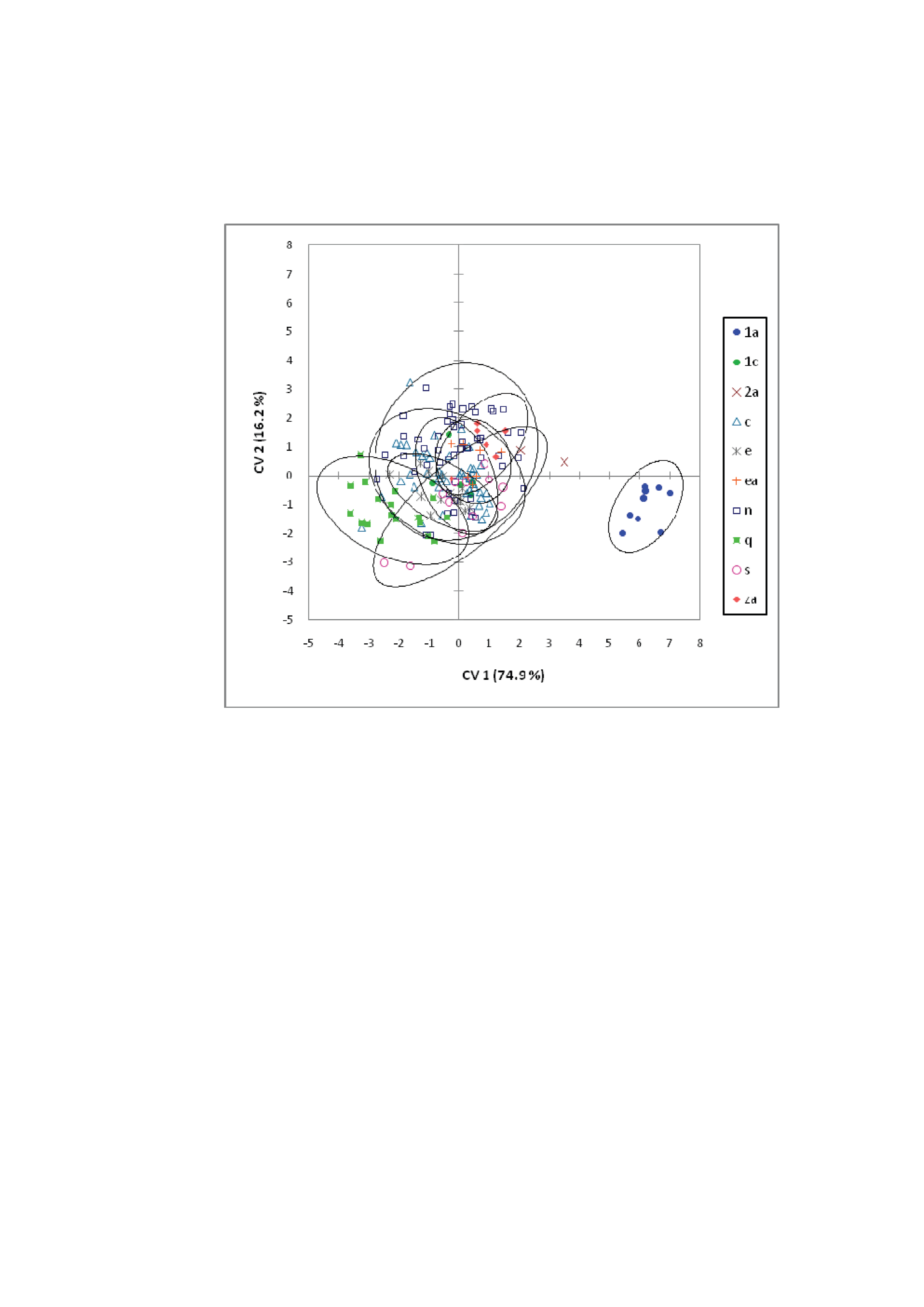

Supplement: Figure S2 — Canonical variates analysis of six craniometric variables in ten Operational Taxonomic Units (OTUs) based on a sample of 171 adult male skulls of R. hildebrandtii and R. eloquens . Clades and lineages defined on molecular grounds indicated by 1a (Clade 1: Mpumalanga Province, SA), 1c (Clade 1: Pafuri, Kruger NP, SA) and 2a (Clade 2). Codes indicate species and OTUs as follows: q = R. eloquens (including type series); balance of OTUs represent R. hildebrandtii: s = southern Zimbabwe; c = central Zimbabwe; e = eastern Zimbabwe; n = northern Zimbabwe; za = Zambia+Malawi; ea = Kenya and Tanzania (including type and co-type from Kenya). 95% confidence ellipses shown for each group. (TIF) [file pone.0041744.s005.tif]

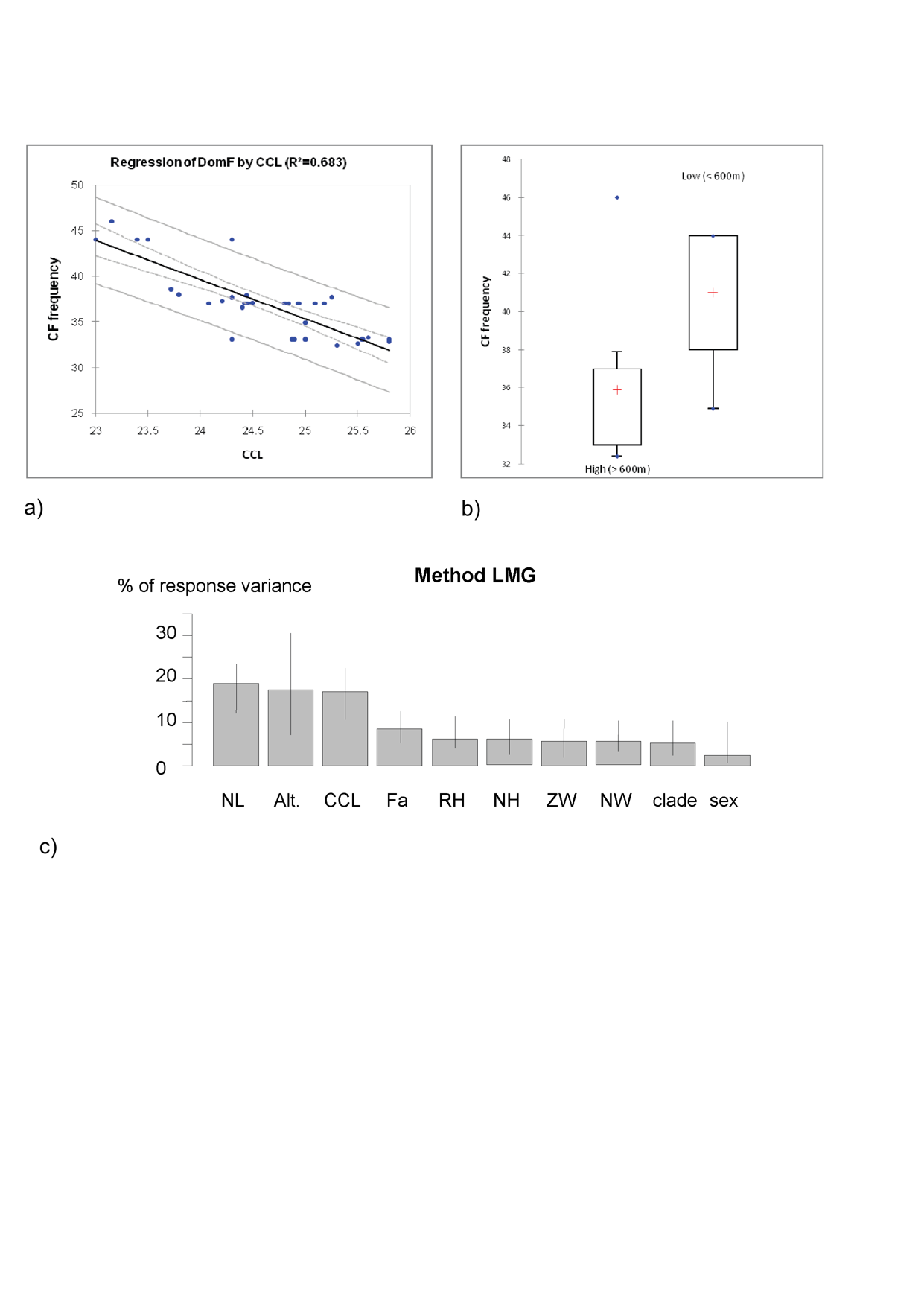

Supplement: Figure S3 — Plots of peak frequency of the CF component of echolocation calls versus condylo-canine skull length (CCL) (a) and altitude (b); and (c) means and 95% confidence limits of relative importance after 1000 bootstrap replications (using “lmg” method in R) for ten predictor variables used in complex linear model (Model 5 in text) to explain variation in CF frequency in R. hildebrandtii . Alt = altitude group (<600 m or >600 m); CCL = condylocanine skull length; FL = forearm length; Clade = membership of molecular clades and subcaldes (Fig. 3); NH = height of rostral chamber; NW = width of rostral chamber; NL = length of skull from occiput to anterior of rostral chamber; RH = relative humidity. (TIF) [file pone.0041744.s006.tif]

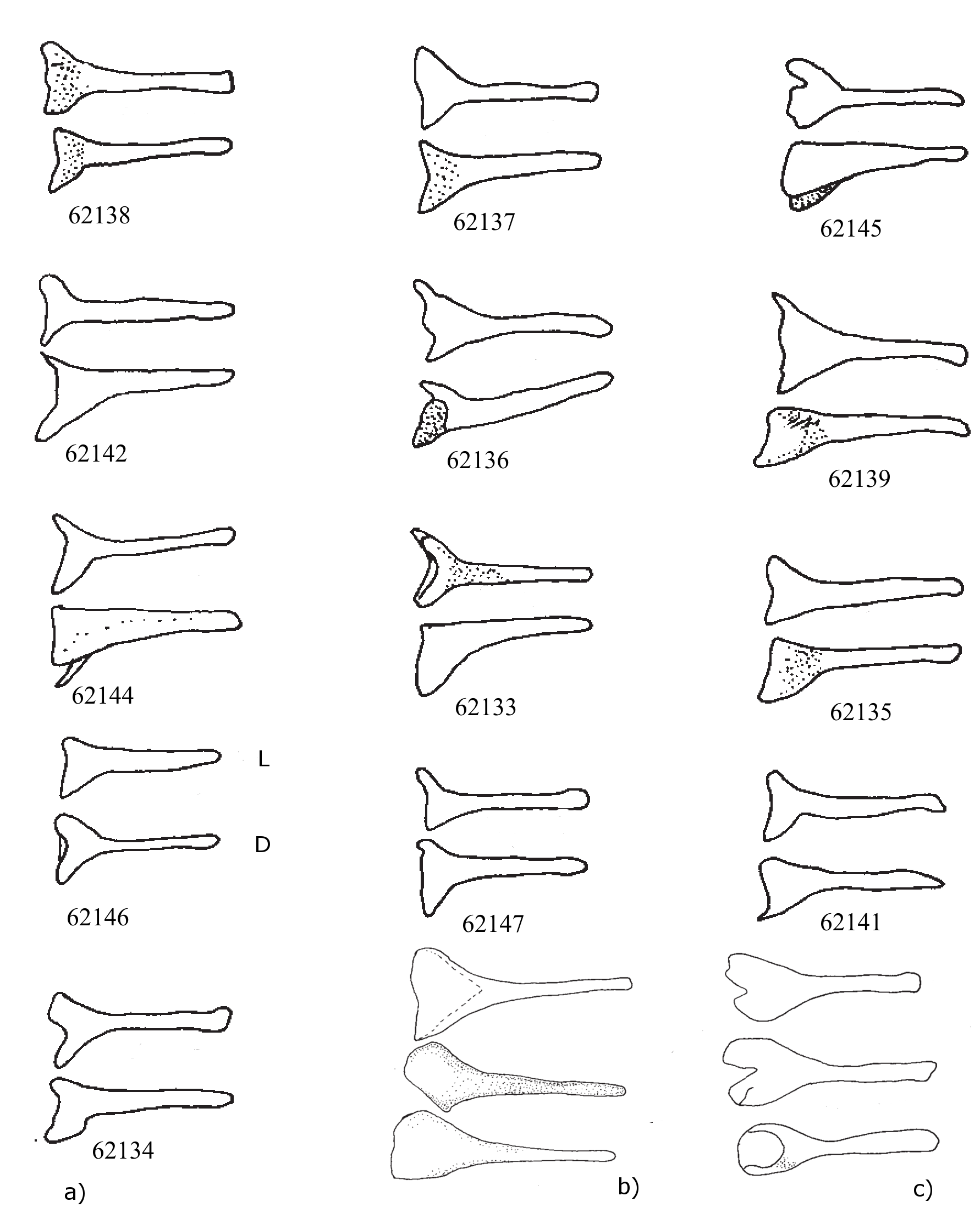

Supplement: Figure S4 — Drawings of bacula from: (a) Marondera, Zimbabwe (NMZB 62133–62139; 62141–62142; 62144–621477; except where noted otherwise, top = dorsal; bottom = lateral), Esigodeni, Zimbabwe (b; NMZB 80237; total length = 4.6 mm) and Luangwa, Zambia (c; NMZB 62149; total length = 2.6 mm). In (b) top = dorsal, middle = lateral and bottom = ventral and (c) top = dorsal, middle = ventral and bottom = lateral. (TIF) [file pone.0041744.s007.tif]
